# Epidemiological factors and mitigation measures influencing production losses in cattle due to bovine viral diarrhoea virus infection: A meta‐analysis

**DOI:** 10.1111/tbed.13300

**Published:** 2019-07-30

**Authors:** Beate Pinior, Sebastien Garcia, Jean J. Minviel, Didier Raboisson

**Affiliations:** ^1^ Institute for Veterinary Public Health University of Veterinary Medicine Vienna Vienna Austria; ^2^ IHAP, INRA, ENVT Université de Toulouse Toulouse France

**Keywords:** biosecurity, economic, epidemiology, eradication, vaccination

## Abstract

Infection with bovine viral diarrhoea virus (BVDV) is associated with a loss in productivity in cattle farms. Determining which factors influence monetary losses due to BVDV could facilitate the implementation of mitigation measures to reduce the burden of BVDV. Mixed‐effect meta‐analysis models were performed to estimate the extent to which the costs of mean annual BVDV production losses per animal may be influenced by epidemiological factors such as BVDV introduction risk, initial prevalence, viral circulation intensity and circulation duration (trial 1). Additionally, changes in mean annual BVDV production losses per animal due to specific mitigation measures (i.e., biosecurity, vaccination, testing and culling, cattle introduction or contact with neighbouring cattle herds) were analysed (trial 2). In total, 19 studies were included in the meta‐analysis to assess mean annual BVDV production losses. The mean annual direct losses were determined to be €42.14 per animal (trial 1). The multivariate meta‐regression showed that four of the previously mentioned epidemiological factors significantly influenced the mean annual BVDV production losses per animal. Indeed, the per animal costs increased to €67.19 when these four factors (trial 1) were considered as “high or moderate” compared to “low”. The meta‐regression analysis revealed that implementation of vaccination and biosecurity measures were associated with an 8%–12% and 28%–29% decrease in BVDV production losses on average, respectively, when simulated herds were compared with or without such mitigation measures (trial 2). This reduction of mean annual BVDV production losses per animal due to mitigation measures was partially counteracted when farmers brought new cattle on to farm or allowed contact with neighbouring cattle herds. The influencing mitigation factors presented here could help to guide farmers in their decision to implement mitigation strategies for the control of BVDV at farm level.

## 1 INTRODUCTION

Bovine viral diarrhoea virus (BVDV) is a *Pestivirus* related to both border disease virus (BDV) and the causative agent of classical swine fever (CSF). BVDV infections have been detected in 88 countries worldwide (Richter et al., 2019) and represent an important infectious disease in the global cattle population (Pinior & Firth, 2017; Scharnböck et al., 2018). Infection causes substantial costs for farmers through increased production losses and mitigation expenditures. Worldwide BVDV production losses have been estimated to be up to 687.80 US dollars (USD) per animal (Richter et al., 2017). Depending on the time and duration of infection, BVDV can cause a considerable number of direct losses, such as morbidity and mortality due to immunosuppression, reduced reproductive performance (e.g., first service conception, extended calving intervals), stillbirth and abortion, congenital deformities and malformations, growth retardation, reduced milk production and average daily weight gain (Burgstaller et al., 2016; Houe, 1999; Marschik et al., 2018; Richter et al., 2017). Mitigation measures may comprise (a) preventing BVDV transmission by control of cattle trade such as testing of cattle before movements and/or reduced replacement rate of cattle possibly carrying persistently infected (PI) foetuses (Houe, Lindberg, & Moennig, 2006), (b) application of a vaccine, (c) biosecurity strategies such as cleaning of equipment, protective clothing, double fencing and (d) general prevention of contact with potential PI animals (Evans et al., 2019) as well as (e) testing and culling to eradicate BVDV. The economic impacts of BVDV for cattle farms have prompted many countries to implement mitigation programmes and the success of these programmes on the reduction of BVDV prevalences in the global cattle population has been reported elsewhere (Scharnböck et al., 2018).

Determination of epidemiological and mitigation influencing factors on BVDV production losses can facilitate the implementation of control and prevention activities regarding BVDV at farm level. The aim of this study was to analyse the extent to which epidemiological factors (e.g., BVDV introduction risk, initial prevalence, viral circulation intensity and BVDV circulation duration) and mitigation measures (e.g., biosecurity, vaccination, testing and culling, cattle introduction or contact with neighbouring cattle herds) may influence the ex‐ante and ex‐post estimated monetary level of production losses due to BVDV infections in the cattle population from the literature.

## 2 MATERIALS AND METHODS

### 2.1 Standardization of collected data

**Figure 1:**
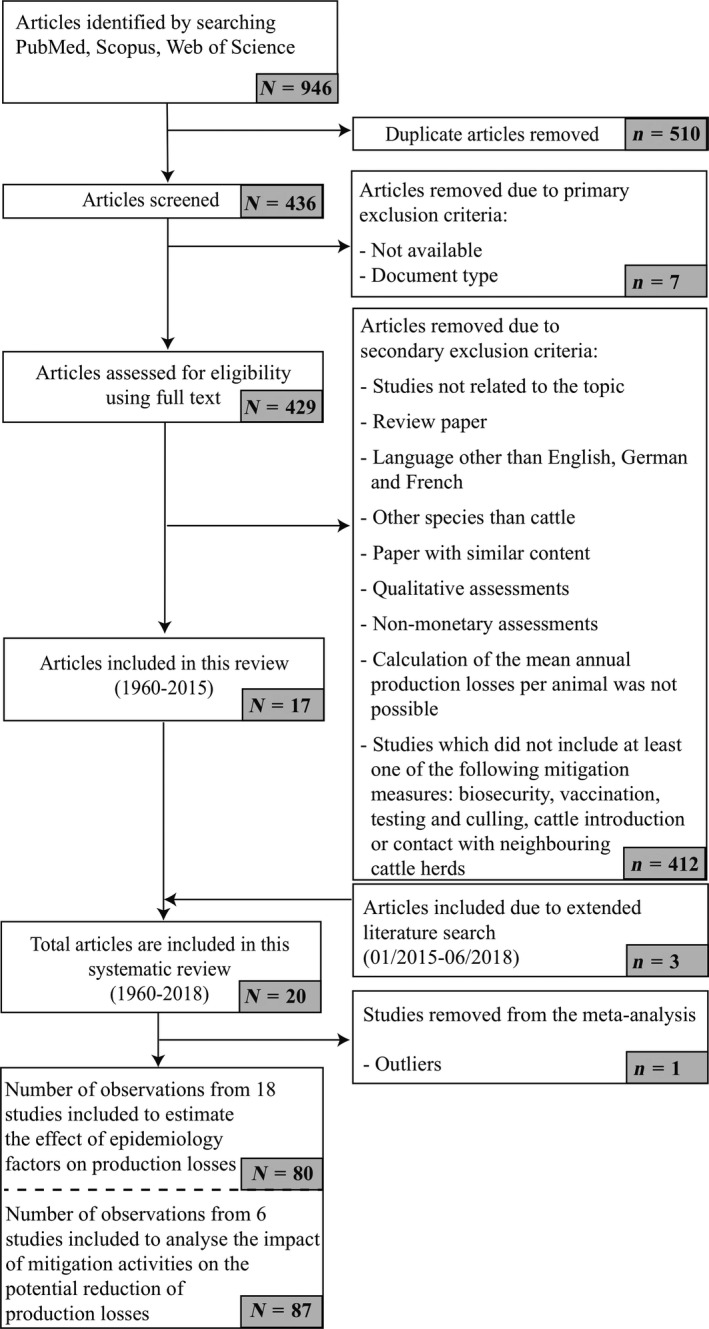
Flow chart of studies incorporated in the systematic review and meta‐analysis

In the present study, we analysed BVDV production losses and covariate data in the period from 1960 to 2015, provided by Pinior et al., (2017) and Richter et al., (2017). The literature research was extended to the period from January 2015 to June 2018 with the following predefined search terms ((bovine viral diarrh* OR bovine virus diarrh* OR bvd OR bvdv) AND (economic* OR financial OR cost*)) in order to identify the greatest possible number of recent publications concerned with the monetary level of BVDV production losses. A search for articles was performed in PubMed, ISI Web of Knowledge, and Scopus. Studies were included in the meta‐analysis if the following criteria were met: (a) studies estimated production losses due to BVDV infections in monetary terms at farm, regional or national level; (b) studies analysed production losses in cattle, i.e., dairy and/or beef (without beef finisher); (c) studies should have published mean (average) annual production losses per animal which covered losses for all cattle in the associated population (even if the animals were not affected, as by Stott et al., (2012)). N.B. no restrictions were defined on the level of monetary losses due to BVDV infections. If studies reported initially losses of only infected cattle, as by Marschik et al., (2018), these losses should be transferable to all cattle in the associated population (infected and uninfected). Consequently, population characteristics such as number of cattle, herd size and in case that losses were published over multiple years, also the time periods of assessments, were essential for the standardization of the losses per animal and per year. Studies for which such standardization was not possible were excluded from further analysis; (d) additionally, studies were included which analysed at least one of the following mitigation strategy: biosecurity, vaccination, testing and culling, cattle introduction or contact with neighbouring cattle herds (Figure 1). N.B. the term “animal” in the presented work covered dairy and/or beef production systems (without beef finisher). The total number of identified publications and the applied two‐step selection process for eligible studies performed in accordance with the PRISMA guidelines (Preferred Reporting Items for Systematic Reviews and Meta‐Analysis), are illustrated in Figure 1. All articles were screened in full by two reviewers (SG, DR) and eligible studies, i.e., which met the inclusion criteria, were then reviewed in full by one reviewer (SG) in accordance with the predefined variables shown in Table 1. All relevant data from the eligible studies were entered into a Microsoft Excel spreadsheet (Table S1–S2). A publication was further divided into different observation sets if the study take different variables according to Table 1 into account and thus published different monetary production losses per animal. For instance, if the study published two different monetary production losses per animal because two different sets of input variables were used, such as high and low transmission rates, then the study was divided into two observations (illustrated as two lines in Table S1 and in the Forest plots). N.B. we considered the fact that these two results from the same study are obtained in closer conditions than results from two separate studies by including “publication” as random factor in our meta‐analysis (see following section: meta‐analysis). The total number of publications included in the presented study is thus not identical to the total number of observations.

**Table 1:** Analysed influencing factors on the estimated mean annual BVDV production losses per animal and summarizing some of these variables into “new built” factors

Analysis criteria	Category	Trial 1		Trial 2	
		Number of observations	Included in the meta‐analysis^c^	Number of observations	Included in the meta‐analysis^c^
General factors					
Study type	Modelling Descriptive^b^	75 5	Y	87 0	Y
Study level	Farm Regional/ National	65 15	Y	81 6	Y
Publication year	Numeric	80	Y	87	Y
Duration (years)	Numeric	80	Y	87	Y
Country	Nominal	80	Y	87	Y
Annual discount rate	Numeric	35	Y	22	Y
Epidemiological factors					
Production system	Dairy Beef Mixed	13 60 7	Y	13 74 0	Y
Number of herds	Numeric	79	Y	–	N
Average herd size	Numeric	75	Y	84	Y(TC_S)
Replacement rate	Numeric	70	Y (B_IR) (B_CI)	87	Y
Management system	Open herds Closed herds	44 36	Y (B_IR)	51 36	Y
In‐calf cow purchase	Yes No	6 10	Y (B_IR) (B_CI)	–	N
Circulation rate	Likelihood Certainty	61 19	Y (B_CI) (B_CD)	34 53	Y
Biosecurity break	By PI By TI No	31 9 2	Y (B_CI)	–	N
Virus circulation at the beginning	PI TI^a^	35 31	Y (B_CI)	–	N
PI prevalence (at animal level)	Numeric	43	Y (B_IR) (B_IP) (B_CI) (B_CD)	–	N
PI prevalence (at herd level)	Numeric	45	Y (B_IR) (B_IP)	–	N
BVDV status at the beginning of the study	Free Infected	30 50	Y (B_IR) (B_IP) (B_CD)	67 20	Y
BVDV spread	Epidemic Endemic Epidemic and then endemic	25 32 23	Y (B_CI) (B_CD)	14 21 52	Y
Transmission rate	Low Moderate High	8 31 41	Y (B_CD)	8 36 43	Y
Contact with neighbouring cattle herds	Yes No	9 51	Y	30 57	Y (BI_S)
Cattle introduction (within herd, %)	0% (unknown) 1%–25% 25%–100% 100%–200%	–	N	57 9 9 9	Y
BVDV introduction risk (B_IR)	1 = Low 2 = Moderate 3 = High	30 20 30	Built used	–	N
BVDV initial prevalence (B_IP)	1 = Low 2 = Moderate 3 = High	6 32 42	Built used	–	N
BVDV viral circulation intensity (B_CI)	1 = Low (endemic) 2 = Moderate (endemic‐epidemic) 3 = High (epidemic)	47 29 4	Built used	–	N
BVDV circulation duration (B_CD)	1 = Low 2 = Moderate 3 = High	30 30 20	Built used	–	N
Mitigation factors					
Biosecurity	Yes No	–	N	55 32	Y (BI_S)
Biosecurity efficacy	Unknown Efficacy = 0%–30% Efficacy = 31%–89% Efficacy = 90%–100%	–	N	58 8 14 7	Y (BI_S)
Biosecurity score (BI_S)	1 = Low 2 = Moderate 3 = High	–	N	32 26 29	Built used
Vaccination	Yes No	–	N	37 50	Y (VA_S)
Vaccination efficacy	Unknown 1 = Efficacy =0%–50% 2 = Efficacy =50%–100%	–	N	50 19 18	Y (VA_S)
Vaccinated population	None Whole herd Heifers and calves Reproductive females	–	N	50 4 19 14	Y
Vaccination frequency	None 2 doses first year then annually Annually	–	N	50 23 14	Y (VA_S)
Vaccination score (VA_S)	1 = Low 2 = Moderate 3 = High	–	N	50 34 3	Built used
Testing and culling	Yes No	–	N	26 61	Y (TC_S)
Testing and culling efficacy	Numeric	–	N	15	Y (TC_S)
Testing and culling score (TC_S)	1 = Low 2 = Moderate 3 = High	–	N	61 4 22	Built used

Abbreviations: B_CD, BVDV circulation duration; B_CI, BVDV viral circulation intensity; B_IP, BVDV initial prevalence; B_IR, BVDV introduction risk; BI_S, Biosecurity score; TC_S, Testing and culling score; VA_S, Vaccination score.

a In contrast to PI animals, which excrete the virus throughout their lives, transient infected (TI) animals excrete BVDV for approximately 14 days.

b Descriptive studies were also included because data about annual production losses per animal and epidemiological and/or mitigation covariates were provided.

c Whether the factor was included in the meta‐analysis is indicated with Y = Yes; N = No; and if the factor was included in the “build used” factor it is indicated with brackets and the abbreviation of the associated build used factor.

Meta‐analysis is used to detect effects across studies by analysing factors (covariate [i.e., independent variable of direct interest such as mitigation measures] or moderators [i.e., study‐level covariates such as characteristics of the study or population]) that may influence the effect. All factors from Table 1 were analysed in the meta‐analysis. However, some factors were only reported in part of the publication (e.g., approximately half of the studies provided PI prevalence values, see Table S1–S2) or the way in which the factors were presented were highly variable among the different publications (e.g., the infected status at the beginning of the studies may have covered seronegative, seropositive, transient infected, persistent infected or immune herds), which may cause bias in the meta‐analysis. To standardize the wide range of published values across the studies and to take the uncertainty within and between the studies into account four new epidemiological factors (i.e., BVDV introduction risk, initial prevalence, viral circulation intensity and circulation duration) and three new mitigation factors (defined as “built used” factors, see Table 1) based on a categorical scale (low, moderate, high) were additionally created, as a combination of data originating from the incorporated studies. An assignment of factors incorporated in the “new build” factors is provided in Table 1, a detailed example of the variable construction is provided in Figure S1 and the associated data are provided in Table S1 and S2. Both the recorded raw data from the literature and the “new build” factors were analysed in the meta‐analysis.

The production losses reported in the literature were standardized per animal and per year. The annual production losses were published in different national currencies and years. A standardization to the Euro (€) and the year 2018 for each respective country was performed as follows:

(1) $$Y\_DL_{\left(€;2018\right)}=\frac{Y\_DL_{X}^{i}}{\tau_{\text{conv}}X_{i\to €}}\times \left(\frac{I\_OCDE_{2018}}{I\_OCDE_{X}}\right)$$

where *Y_DL* (*€*; 2018) represents the annual mean BVDV production losses per animal in € in 2018 and *i* indicates the national currency of the respective country for which production losses were determined in the year *X*. The nominal exchange rate ($\tau_{\text{conv}}X_{i\to €}$) was distinguished between the Eurozone (i.e., exchange rate of the national currency *i* into the currency € in 2002) and non‐Eurozone (i.e., exchange rate of the national currency *i* into the currency € in the year of publication). The index *I_OCDEx* includes the economic annual growth rate of the respective country and incorporates the inflation rate based on the consumer price index.

### 2.2 Meta‐analysis

The outcome variable in trial 1, which included the situation before any mitigation measure had been taken, is presented by the mean annual production losses per animal. The general and epidemiological factors (Table 1) were used as explanatory variables for the mean annual production losses per animal in the meta‐regression analysis. In trial 2, the outcome variable is shown as the percentage decrease in the mean annual production losses per animal. Here, the effect of the recorded mitigation measures (Table 1) on the changes in BVDV production losses from the literature was investigated and the changes were expressed as the percentage difference of the production losses before and after implemented mitigation measures.

Trials 1 and 2 were performed independently in a random‐effect meta‐analysis model (without factors from Table 1 but with publication as random factor, i.e. if multiple data points were collected for a variable  from one study (repeated measures), “publication” might be thought as random factor and is analogous to random effects in classical ANOVA) and mixed‐effects model (with factors from Table 1 and with publication as random factor). In the first step, the heterogeneity of the incorporated studies in the meta‐analysis was determined as follows: (a) calculating the percentage of total variation across the studies by estimation of the Higgins inverse variance (*I* ^2^) index (lay between 0% and 100%), whereby I^2^ greater than 50% indicated substantial heterogeneity between studies and (b) calculating the degree of between study variance, i.e., the Cochran's *Q*‐Test where *p* < .05 indicated heterogeneity. The limitation of *I* ^2^ and Cochran's *Q*‐Test is that both provide only a value of the total heterogeneity between the considered studies in the meta‐analysis but no information about the factors which causing the potentially heterogeneity. If evidence of high variability between studies was determined, the next step was to perform a meta‐regression, i.e., quantification of heterogeneity in effect size among studies by including factors (covariate or moderators), referred to as mixed‐effect model (Viechtbauer, 2010). The inclusion of the factors was conducted as follows: univariate meta‐regressions were first performed to identify general, epidemiological and mitigation factors according to Table 1 that may have had a significant association with the mean annual BVDV production losses per animal. Any significant factors in the univariate test was selected as a potential influencing factor for the multivariate analysis. The multivariate meta‐regression was based on the following selection process: adding stepwise single significant factor of the univariate regression (significance was declared at *p* ≤ .05) in the multivariate model (Bursac, Gauss, Williams, & Hosmer, 2008), followed by removing correlated and non‐significant factors from the multivariate model, identification of significant factors combination that reduce the Akaike Information Criteria (AIC) and increasing the value of r square (*R* ^2^), as well as reducing the heterogeneity between the included studies in the meta‐analysis. The *τ* ^2^ (residual heterogeneity variance) denoted the amount of the heterogeneity that may have not explained through the inclusion of the factors in the meta‐analysis. A reference class for each factor was chosen to allow a comparison of the effect size (Table 2).

**Table 2:** Final multivariate‐meta‐regression results of the epidemiological and mitigation factors influencing estimated mean annual BVDV production losses per animal

	Estimate coefficient	Standard error	*Z*‐value	*p*‐value	95% CI
Model 1: Epidemiological factors					
Intercept	0.13	6.18	0.02	.9800	11.97/12.23
BVDV introduction risk[Link]	34.33	0.90	38.15	<.0001	32.57/36.10
BVDV initial prevalence[Link]	7.31	0.56	13.17	<.0001	6.22/8.40
BVDV viral circulation intensity[Link]	31.74	0.89	35.58	<.0001	29.99/33.49
BVDV circulation duration[Link]	4.30	0.14	30.59	<.0001	4.02/4.58
Introduction risk: initial prevalence	−10.49	1.01	−10.40	<.0001	−12.47/−8.51
Model 2: Mitigation factors					
Intercept	0.44	0.08	5.16	<.0001	0.28/0.62
Vaccination[Link]	0.08	0.01	5.52	<.0001	0.05/0.11
Biosecurity[Link]	0.29	0.01	25.17	<.0001	0.27/0.32
Cattle introduction (No)	ref				
1%–25% Introduction[Link]	−0.24	0.01	−18.69	<.0001	−0.27/−0.22
25%–100% Introduction[Link]	−0.25	0.01	−15.76	<.0001	−0.29/−0.23
100%–200% Introduction[Link]	−0.03	0.01	−2.08	.0300	−0.06/0.00
Model 3: Mitigation factors					
Intercept	0.34	0.10	3.32	.0009	0.14/0.55
Vaccination[Link]	0.12	0.01	9.13	<.0001	0.10/0.15
Biosecurity[Link]	0.28	0.01	24.19	<.0001	0.26/0.31
Contact with neighbouring cattle herds[Link]	−0.18	0.00	−18.74	<.0001	−0.19/−0.16

For classes “moderate to high” compared to reference class “low.”

For class “yes” compared to reference class “no.”

% of cattle introduced in the herd compared to the total herd size of the farm.

Publication bias was identified by performing the Egger‐test, a regression test for funnel plot asymmetry and inspection of the associated funnel plots. An influential case diagnostic (i.e., DFFITS value, Cook's distances, covariance ratios, estimates of *τ* ^2^ and test statistics for [residual] heterogeneity) was performed to identify outliers. For final meta‐regressions, the mean annual BVDV production losses per animal (trial 1) and the percentage difference of production losses before and after implementation of mitigation measures (trial 2) and their respective 95% confidence intervals (CIs) are shown in the forest plots, stratified by the final epidemiological (trial 1) and mitigation (trial 2) factors. The meta‐analyses were implemented in R (Version 3.4.1 R Foundation for Statistical Computing) using the Metafor package (Viechtbauer, 2010).

## 3 RESULTS

### 3.1 Trial 1

**Figure 2:**
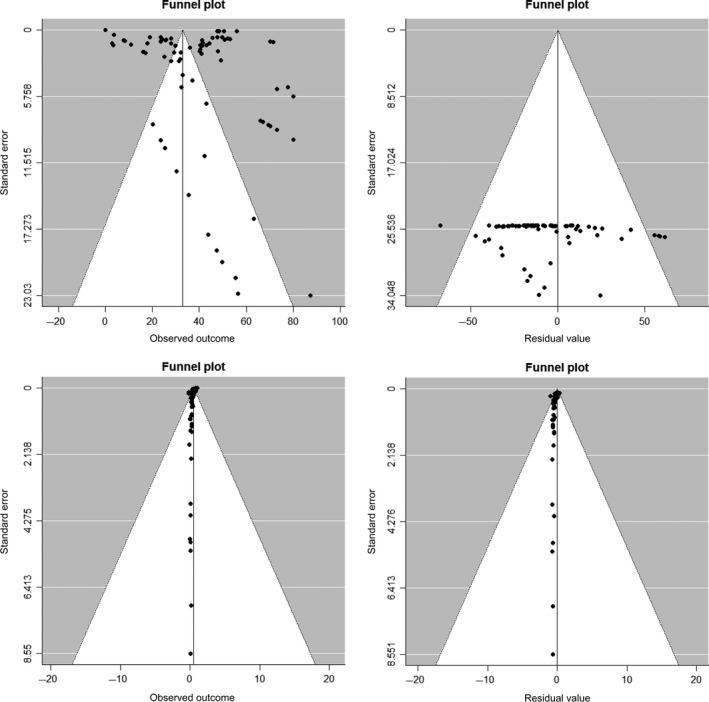
Funnel Plot of the random meta‐analysis of studies without (a) incorporating of epidemiological and (b) mitigation factors (see left side of the Figure); mixed‐effect meta‐analysis of studies with incorporated (a) epidemiological and (b) mitigation factors (see right side of the Figure)

In total, 20 studies were included in the meta‐analysis. Trials 1 and 2 included 19 and 6 publications with 83 and 87 observations, respectively. The influential case diagnostic of trial 1 indicated three observations and one study as sources of asymmetry (Figure S2; Table S3). These observations were considerably higher regarding production losses (with a mean of €215) compared to other observations (with a mean of €40) and thus highly influence the results of the meta‐regression. Consequently, three observations and one study from trial 1 were excluded in the present meta‐analysis (Figure 1). The funnel plot with 80 observations covering 18 studies (trial 1) did not show any asymmetry issues for the incorporated studies (Figure 2), despite the existence of many outliers. A publication bias was determined with the Egger's test (*z* = 5.4616, *p* < .0001). The heterogeneity between the studies was very high (*I* ^2^ = 99.93%; AIC = 12,301; *Q*‐Test: *x* ^2^ = 32,931; *df* = 78; *p* < .001).

**Figure 3:**
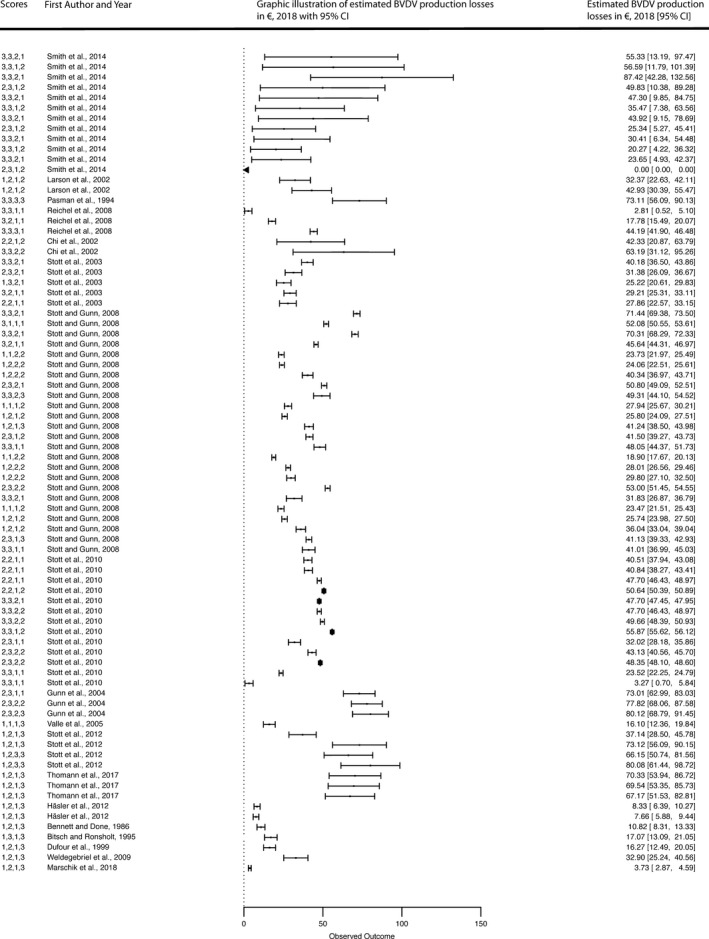
Forest plot of the meta‐analysis models including the significant epidemiological factors (“build used” factors of Table 1). The column on the right refers to the mean annual BVDV production losses per animal with the corresponding confidence intervals (shown in brackets). The different single numbers attached before the authors' names (left column) represent the “build used” factors of Table 1. The numbers represent the following scores, i.e., 1 = low; 2 = moderate and 3 = high. The order of the numbers can be classified as follows: first number covered BVDV introduction risk, followed by initial prevalence, circulation intensity and duration (see also Table S1). N.B. the forest plot may include the same combination of numbers more than one before the authors’ names within a study because different sets of input parameters were used, resulting in different estimated mean annual production losses per animal. The grey diamonds represent the effect size adjusted for the “build used” epidemiological factors. Forest plots of mitigation measures are provided in Supplementary Figure S5–S6. N.B. Full references of authors shown in the forest plots are available in Supplementary Table S4

The random meta‐regression without factor of Table 1 indicated a mean annual production loss per animal of €42.14 (se = 8.83; *p* < .001). This value represents the estimated mean annual production loss per animal across the studies and the estimated mean annual production losses per study is shown in Figure 3. In the univariate meta‐regressions, BVDV production losses were associated with the factors production system, BVDV introduction risk, initial prevalence, viral circulation intensity and circulation duration. The other general and epidemiological variables in Table 1 were not statistically associated with mean annual BVDV production losses per animal. In the multivariate mixed‐effect regression (trial 1), the mean annual BVDV production losses per animal were also significantly associated with the BVDV introduction risk, initial prevalence, viral circulation intensity and circulation duration (Table 2). The mean annual BVDV production losses per animal were €34.33 and €31.74 higher, respectively, if the BVDV introduction risk and the BVDV viral circulation intensity were high, compared to studies with lower risk values. The mean annual production losses per animal reached up to €67.19 (i.e., 34.33 + 7.31 + 31.74 + 4.30–10.49) for a situation where all four statistically significant factors were presented (Table 2).

### 3.2 Trial 2

In trial 2, the funnel plot did not show any asymmetry issues for the incorporated studies (Figure 2), despite a few observations having very high standard errors. Specifically, the influential case diagnostic indicated 2 outliers for both mitigation models (i.e., with cattle introduction or with contact with neighbouring cattle herds; Figure S3–S4) but exclusion did not change the significant associations or the coefficient observed in the final meta‐regressions, which have been kept as the final ones. A publication bias was reported by the Egger's test (z = 6.4536, *p* < .0001). The heterogeneity of the dataset was high (*I* ^2^ = 98.55%; AIC = 2,179; *Q*‐test: *x* ^2^ = 2,357; *df* = 80; *p* < .001). In the univariate meta‐regressions, the mitigation factors (i.e., biosecurity, vaccination, testing and culling and contact with neighbouring cattle herds) were statistically associated with BVDV production losses. In the multivariate mixed‐effect meta‐regressions, biosecurity, vaccination and cattle introduction or contact with neighbouring cattle herds were significantly associated with the change in the BVDV production losses (Table 2), whereas testing and culling was not identified as a significant factor. Implementation of vaccination and biosecurity measures were on average associated with an 8%–12% and 28%–29% decrease in BVDV production losses, respectively, when simulated herds from the literature were compared with or without such mitigation measures. This reduction of BVDV production losses per animal due to mitigation measures was partially counteracted on average by 18% when farmers brought new cattle on to farm (cattle introduction) or allowed contact with neighbouring cattle herds (Table 2). The percentage difference of the production losses before and after implemented mitigation measures per study is shown in the Figure S5–S6.

## 4 DISCUSSION

In order to analyse previously published studies with a specific emphasis on production losses incurred by BVDV infection, we reviewed 436 articles in full, of which 19 different articles were considered for detailed analysis. These 19 studies published general, epidemiological and mitigation factors with regard to BVDV production losses. Until now, there has been no systematic review or meta‐analysis investigating the extent to which the costs of BVDV production losses may be influenced by epidemiological factors and mitigation measures. In contrast to a systematic review, a meta‐analysis attempts to accurately summarize results across different publications and investigate factors (covariate or moderators) that influence the outcome variable (Gurevitch, Koricheva, Nakagawa, & Stewart, 2018). In the presented study, the primary goal was to estimate the effects of epidemiological factors and mitigation activities that may influence the mean annual monetary BVDV production losses per animal, as published in the literature.

Over all studies from the literature (see Table S4), we calculated a mean annual BVDV production loss of €42.14 per animal (without factors; random‐effect meta‐analysis model). This estimated mean annual production loss per animal covered infected and uninfected animals and is probably lower than if only losses from infected cattle would be taken into account. Our meta‐analysis demonstrated that studies which assumed a high BVDV introduction risk, initial prevalence, viral circulation intensity and circulation duration, compared to low, significantly increased the mean annual BVDV production losses to €67.19 per animal (Table 2). For instance, in the present work, it was shown that across all studies incorporated in the meta‐analysis, the mean annual BVDV production losses per animal were €34.33 higher in cattle herds with a high simulated introduction risk compared to herds with a low risk (Table 2). It is well recognized that the introduction of new cattle into a herd is one of the most important factor for the transmission of BVDV. Many existing analyses in the literature have indicated that the purchase of animals, in particular pregnant cows, increase the chance for BVDV introduction into a fully susceptible herd (such as Bitsch, Hansen, & Rønsholt, 2000; Santman‐Berends, Mars, Duijn, Broek, & Schaik, 2017) and consequently influence the economic impact of BVDV.

Additionally, contact with neighbouring cattle herds or common housing of ruminants should be considered as an important factor for virus transmission and hence for BVDV production losses (Graham et al., 2016; Kaiser, Nebel, Schüpbach‐Regula, Zanoni, & Schweizer, 2017). Our analysis emphasizes that reduction of mean annual BVDV production losses per animal due to mitigation measures was partially counteracted on average by 18% when farmers introduced new cattle into a farm (cattle introduction) or allowed contact with neighbouring cattle herds, compared to farmer without introduction of cattle or contact to other herds (Table 2). A limitation regarding the recorded initial prevalence of BVDV, introduction risk and recorded contact with neighbouring cattle herds is that we do not taken into account the inter‐species transmission of *Pestivirus* from the literature because the majority of studies neglected to mention it. The number of cattle herds considered in the analysed studies was not identified as a relevant factor explaining the mean annual BVDV production losses per animal. The main reason for this is that the majority of analysed studies considered a fixed number of herds and herd size when simulating the introduction of BVDV with or without mitigation measures. Individual studies, such as by Stott et al., (2012), identified twice higher mean annual BVDV production losses per animal in dairy than in beef herds. Nonetheless, over all incorporated studies in the meta‐analysis, the factor “production system” was not determined to be a significant factor on mean annual BVDV production losses per animal. One reason could be that some of the considered studies in this meta‐analysis estimate production losses for beef herds based on observations in dairy herds, when no observation for beef herds were available (e.g., in the study by Valle et al., (2005)).

The present study confirms the success of mitigation activities with regard to reduction in mean annual BVDV production losses per animal. Mean annual BVDV direct losses per animal were 8%–12% and 28%–29% lower in studies including vaccination and biosecurity as compared to studies omitting these mitigation measures, respectively (Table 2). This result is in agreement with the meta‐analysis by Newcomer, Walz, Givens, and Wilson (2015). The study reveals that abortion decreased by 45% and the foetal infection rate decreased by approximately 85% in cattle herds vaccinated against BVDV compared with non‐vaccinated herds (Newcomer et al., 2015). In contrast to vaccination, biosecurity reduces BVDV production losses more effectively (Table 2). This may be related to the fact that farmers often fail to apply the vaccine correctly, vaccines are not proven to be fully protective (Evans et al., 2019), e.g., in the prevention of in‐utero transmission of the virus (Moennig & Brownlie, 2001), the BVDV vaccine does not provide life‐long immunity and hence periodic vaccination is required (Weldegebriel, Gunn, & Stott, 2009), live BVDV vaccine could be contaminated with other viruses (Lindberg, 2003), and/or a critical vaccination coverage rate should be reached to prevent new PI animals (Scharnböck et al., 2018). In the present multivariate‐regression, testing and culling was not identified as a significant factor in changing the mean annual production losses per animal due to BVDV infection. The fact that a low number of studies incorporated in the present study have analysed the effect of culling strategies on BVDV production losses (*n* = 26 out of 87 observations; see Table 1) may have contributed to culling measures being not statistically significant. Another reason for this is that Pasman, Dijkhuizen, and Wentink (1994) showed that a testing and culling strategy was not justified as an economical strategy, if reinfection in cattle herds had occurred. Thus, the success of the culling strategy on the reduction of BVDV production losses depends on future re‐infections. The latter could not be incorporated as a factor in the presented meta‐analysis because most of the studies neglected to distinguish between infection and re‐infections. A further reason could be that the time point of implementation and thus the aim of eradication differs between vaccination and biosecurity. While eradication activities reduced the future profit of animal owners due to the premature culling of animals and increased the natural immunity of herds, vaccination and biosecurity prevented incursion and/or (re)‐introduction of BVDV. Nonetheless, Scharnböck et al., (2018) showed that eradication activities reduce the virus transmission within and between herds and thus culling strategies can successfully reduce the global BVDV prevalences over time.

Our meta‐analysis demonstrated a large heterogeneity and publication bias across the identified studies. The heterogeneity of epidemiological and economic studies has already been noted in other published studies regarding BVDV (Pinior et al., 2017; Richter et al., 2017; Scharnböck et al., 2018). Decreasing heterogeneity substantially required (a) the standardization of the outcome variable (as done in the presented study by discounting adjustments of the monetary level of direct losses and by consideration of published mean annual production losses per animal of both infected and uninfected cattle in the population), (b) stepwise inclusion of factors (as done in the presented study in the univariate and multivariate meta‐regression based on e.g., *I* ^2^, AIC, *R* ^2^) and (c) standardization of factors to improve the comparison between the incorporated studies (as done in the presented study by creating new epidemiological and mitigation factors). This latter procedure may have created additional bias by combining the variables into few new factors. The difficulty faced in reducing heterogeneity in the present work suggests that other covariates not taken into account may contribute to the heterogeneity and identified outliers. For instance, the herd immunity, improvements of the breeding performance over the time, period of gestation, management practices, age of animals, duration of mitigation activities, different BVDV status, level of herd production, stocking density, community pasturing activities, case‐selection procedure, virulence of the infecting BVDV genotype or strain (Hessman et al., 2009; Houe, 1999; Scharnböck et al., 2018). Different modelling approaches, study assumptions, input parameters and the unbalanced number of studies identified for some factors may have also contributed to the high heterogeneity between the studies presented here. A range of different methods were applied in the literature, such as stochastic simulation models (in 12 of the 19 studies), deterministic models (*n* = 4) such as decision trees, and other methods (*n* = 3). In total, five studies (Stott & Gunn, 2008; Stott, Humphry, & Gunn, 2010; Stott et al., 2012; Stott, Lloyd, Humphry, & Gunn, 2003; Weldegebriel et al., 2009) used the same stochastic simulation approach, developed by Gunn, Stott, and Humphry (2004). All of these studies considered four disease states (susceptible, transiently infected, immune and/or persistent infection), constant herd size with animal movements (replacement or death), naïve herds at the beginning of the simulation, initial source of BVDV introduction due to contact with neighbouring herd or introduction of BVDV infected animals, and different annual transmission rates, such as the probability assumed of infectious contact between a susceptible and a PI animal.

Although the modelling approach and the estimated input parameters used in these five studies are closely followed that of Gunn et al., (2004), the following model modifications were incorporated which may influence the apparent spread of the animal disease and the economic impact of BVDV: constant herd size varied between all studies (ranged from 14 to 230 head) which may influence the basis reproduction number (*R*_0_); Stott et al., (2003) and Stott and Gunn (2008) considered not only naïve herds at the beginning of the simulation but also herds with unknown BVDV status. This modification lead to the simplification in which animals were allocated randomly to the disease states to fulfil the assumed fixed antibody (positive) prevalence at a herd level of 0.95 and antigen (positive) prevalence of 0.50. Furthermore, the five studies considered the probability of successfully avoiding contact instead of the probability of infectious contact between a susceptible and a PI animal. The rate of avoiding infection taken into account in the literature differs slightly, e.g. Stott et al., (2010) incorporated a larger range of probabilities regarding the contact aversion between the animals as compared to Stott et al., (2003). Additionally, the mitigation options, i.e. biosecurity and/or vaccination (vaccination efficacy ranged from 60% to 90%) were considered by Stott and Gunn (2008) and Stott et al., (2012), while Gunn et al., (2004) assume no mitigation and no re‐infection with the disease. The latter differs also from the work by Stott et al., (2010), who analysed the potential impact of re‐infection on the BVDV production losses. Weldegebriel et al., (2009) and Stott et al., (2012) adapted the model by Gunn et al., (2004) to suit dairy herds rather than only beef suckler herds and therefore the input parameters varied regarding the assumed replacement rate (changed from 15% to 30%), model time steps (changed from yearly to quarterly period in order to reflect the seasonal milk production cycle), prevalences, economic parameters and the slightly different probability of biosecurity breakdown in any year of the 10‐year simulated epidemic. All these differences between the original study by Gunn et al., (2004) and the other five studies may explain the wide range of mean annual production losses per animal (ranging from €2.50 to €69.00; mean: €29.19) reported.

However, when these six studies were compared with the other incorporated studies (*n* = 13, see Table S4) which do not use the stochastic model by Gunn et al., (2004), the following differences were identified: the mean annual production losses per animal was approximately €7.00 higher; different ex‐ante or ex‐post methods were used, while the studies based on the model by Gunn et al., (2004) are all prediction studies; varying assumptions about the effectiveness of mitigation options (e.g., some studies assumed 100% efficacy of vaccine which caused no spread of BVDV and thus no production losses; Santman‐Berends, Mars, Duijn, & Schaik, 2015), and about the transmission probabilities. For instance, one study assumed that 60% of all birth of PI animals in the herds will not lead to losses from BVDV infections (Santman‐Berends et al., 2015) or other studies taken a constant transmission rate into account (Pasman et al., 1994), or neglected infection of naïve herds, or production losses by infected calves, youngstock, or transiently‐infected animals such as by Pasman et al., (1994), Reichel, Hill, and Voges (2008), Santman‐Berends et al., (2015), Thomann et al., (2017) and Marschik et al., (2018). The latter could lead to underestimation of the true economic impact. Furthermore, the majority of the studies include variability of values by incorporating PERT distributions of herd size, prevalences, economic values or incorporating time‐dependent variability into the model, i.e., the average time for a herd in a disease state changed during the simulations, while the other six studies are based on the model by Gunn et al., (2004) taken largely constant epidemiology and economic values into account. Further, the majority of the studies assume different levels of discounting rates and the higher the discounting rate, the lower the level of the current production losses in the studies. The six studies based on the model by Gunn et al., (2004) took immunosuppression into account, whereas the other studies (such as the study by Thomann et al., (2017) and Marschik et al., (2018)) did not use the BVDV model by Gunn et al., (2004) neglected it. Further difference between applied BVDV models are given in the review article by Viet, Fourichon, and Seegers (2007). The variation of the level and type of input variables between the studies can be justified because the true economic impact of BVDV infection in a population is often unknown. Large epidemiological studies and detailed documentation of production data are rare. Richter et al., (2017) demonstrated that production losses vary considerably in the literature due to uncertainty and knowledge gaps regarding the epidemiology of BVDV infections (Evans et al., 2019). Although different epidemiology and mitigation situations were taken into account, the estimated mean annual production losses per animal in this work can only give rough indications of the true annual economic impact of BVDV on production due to the uncertainty of how representative individual studies actually are for the whole cattle population. Despite the existence of such limitations, pooling data from numerous studies and countries is helpful as it provides a more general overview of the influencing factors and is more powerful and less biased than any individual study or conventional methods (Gurevitch et al., 2018; Scharnböck et al., 2018). Thus, the results of the presented study could be used to increase awareness of factors influencing mean annual BVDV production losses per animal and to support decision‐making by farmers and veterinary authorities implementing mitigation measures such as biosecurity measures or control of cattle introduction against BVDV. The latter is particularly essential for cattle owners located in BVDV‐free regions to guarantee their freedom from an animal disease that is not globally regulated. BVDV‐free countries with a fully susceptible population will have a higher BVDV introduction risk and thus higher impact on production compared to countries with a high proportion of seropositive cattle. Nonetheless, the monetary benefit of mitigation strategies, such as biosecurity, will be highly variable due to (a) wide range of determined annual production losses per animal (Figure 3), (b) varying degrees of implementation by the farmers and (c) different epidemiological circumstances such as infection status of the area surrounding the farm.

## 5 CONCLUSIONS

The mean annual production losses due to BVDV infection was found to be €42.14 per animal. The costs increased to €67.19 when the BVDV introduction risk, initial prevalence, viral circulation intensity and circulation duration were “high or moderate” compared to “low.” Our results reveal that the implementation of vaccination and biosecurity measures was associated with an 8%–12% and 28%–29% decrease in mean annual BVDV production losses on average, respectively, when simulated herds with or without such mitigation measures were compared. This reduction of BVDV production losses per animal due to mitigation measures was partially counteracted on average by 18% when farmers introduced new cattle into a farm or allowed contact with neighbouring cattle herds.

## CONFLICT OF INTEREST

None of the authors of this paper has a financial or personal relationship with other people or organizations that could inappropriately influence or bias the content of the paper.

## ETHICAL APPROVAL

Ethical Statement is not applicable because the manuscript is a systematic review of the literature.
