# Supplementary material for: Epidemiological factors and mitigation measures influencing production losses in cattle due to bovine viral diarrhoea virus infection: A meta‐analysis
Source: Transbound Emerg Dis. 2019 Jul 30;66(6):2426–39. doi: 10.1111/tbed.13300 (PMC6900039; doi:10.1111/tbed.13300)
Supplement: Supplementary file 1 [file TBED-66-2426-s001.docx]

**Supplementary Material**

**Epidemiological factors and mitigation measures influencing production losses in cattle due to bovine viral diarrhoea virus (BVDV) infection: a meta-analysis**

Beate Pinior^a^, Sébastien Garcia^b^, Jean Joseph Minviel^b^, Didier Raboisson^b^

*^a^ Institute for Veterinary Public Health, University of Veterinary Medicine Vienna, Veterinärplatz 1, 1210 Vienna, Austria*

*^b^ IHAP, Université de Toulouse, INRA, ENVT, 23 chemin des Capelles, 31076 Toulouse, France*

**Figure legends**

**Figure S1:** An example of the variable construction of the three “new build” mitigation factors a) biosecurity score, vaccination score and testing and culling score (trial 2).

**Figure S2:** Influential case diagnostic and the identified outliers (shown as red circles) in the meta-regression from trial 1 (annual mean BVDV production losses). The associated abbreviation list is shown in Table S3.

**Figure S3:** Influential case diagnostic and the identified outliers (shown as red circles) in the meta-regression from trial 2, model 2 i.e. with biosecurity, vaccination and cattle introduction (change in BVDV production losses). The associated abbreviation list is shown in Table S3.

**Figure S4:** Influential case diagnostic and the identified outliers (shown as red circles) in the meta-regression from trial 2, model 3 i.e. with biosecurity, vaccination and contact with (neighbouring) cattle herds (change in BVDV production losses). The associated abbreviation list is shown in Table S3.

**Figure S5:** Forest plot of studies covering biosecurity, vaccination and cattle introduction. The column on the right refers to the percentage difference of the production losses before and after mitigation measures with the corresponding confidence intervals (shown in brackets). A positive percentage value indicated a benefit and negative value shows additional losses due to implemented mitigation measures. The different single letters attached before the authors’ names (left column) represent the following classification, i.e. Y=Yes; N=No and the numbers show the proportion of introduced cattle compared to the original herd size, i.e., 0.25 indicated that 25% of the cattle were replaced compared to original herd size (see Table 1). The order of the letters follows the assignment biosecurity, vaccination and cattle introduction (see also Table S2). The grey diamonds represent the effect size adjusted for the biosecurity, vaccination and cattle introduction. N.B. Full references of authors shown in the forest plots are available in the Supplementary Table S4.

**Figure S6:** Forest plot of studies covering vaccination, biosecurity and contact with (neighbouring) cattle herds. The column on the right refers to the percentage difference of the production losses before and after mitigation measures with the corresponding confidence intervals (shown in brackets). A positive percentage value indicated a benefit and negative value shows additional losses due to implemented mitigation measures. The different single letters attached before the authors’ names (left column) represent the following classification, i.e. Y=Yes; N=No. The order of the letters follows the assignment vaccination, biosecurity and contact with (neighbouring) cattle herds (see also Table S2). The grey diamonds represent the effect size adjusted for vaccination, biosecurity and contact with (neighbouring) cattle herds. N.B. Full references of authors shown in the forest plots are available in the Supplementary Table S4.

**Table legend**

**Table S1:** Data used for the meta-analysis of the trial 1. New built data originating from studies are highlighted in light grey.

**Table S2:** Data used for the meta-analysis of the trial 2. New built data originating from studies are highlighted in light grey.

**Table S3:** Abbreviation list of the influence case diagnostic shown in Figure S2-S4.

**Table S4:** Full references of the considered studies in the final meta-regression analysis.

**Figure S1:** An example of the variable construction of the three “new build” mitigation factors a) biosecurity score, vaccination score and testing and culling score (trial 2).

**Figure S2:** Influential case diagnostic and the identified outliers (shown as red circles) in the meta-regression from trial 1 (annual mean BVDV production losses). The associated abbreviation list is shown in Table S3.

**
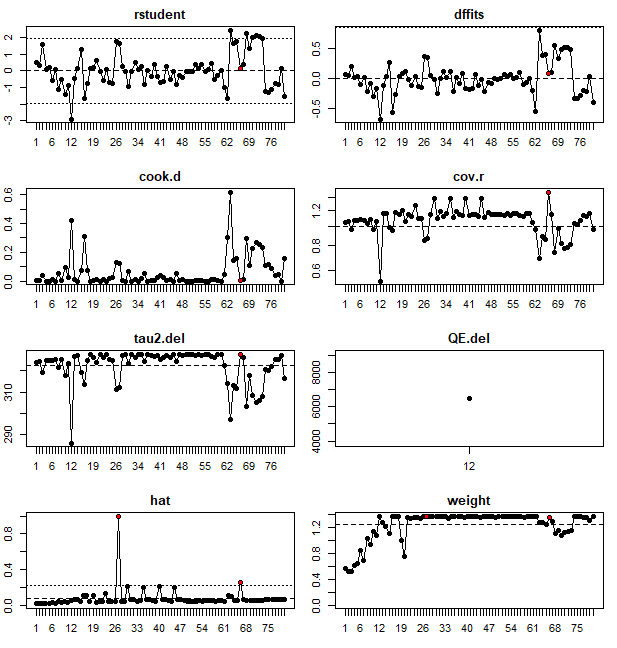
**

**Figure S3:** Influential case diagnostic and the identified outliers (shown as red circles) in the meta-regression from trial 2, model 2 i.e. with biosecurity, vaccination and cattle introduction (change in BVDV production losses). The associated abbreviation list is shown in Table S3.


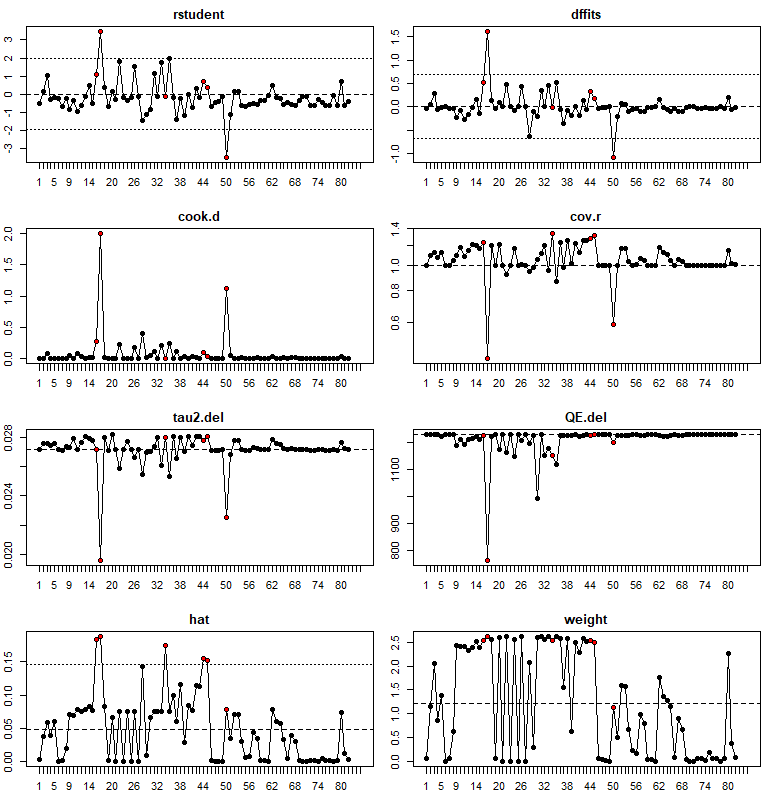


**Figure S4:** Influential case diagnostic and the identified outliers (shown as red circles) in the meta-regression from trial 2, model 3 i.e. with biosecurity, vaccination and contact with (neighbouring) cattle herds (change in BVDV production losses). The associated abbreviation list is shown in Table S3.


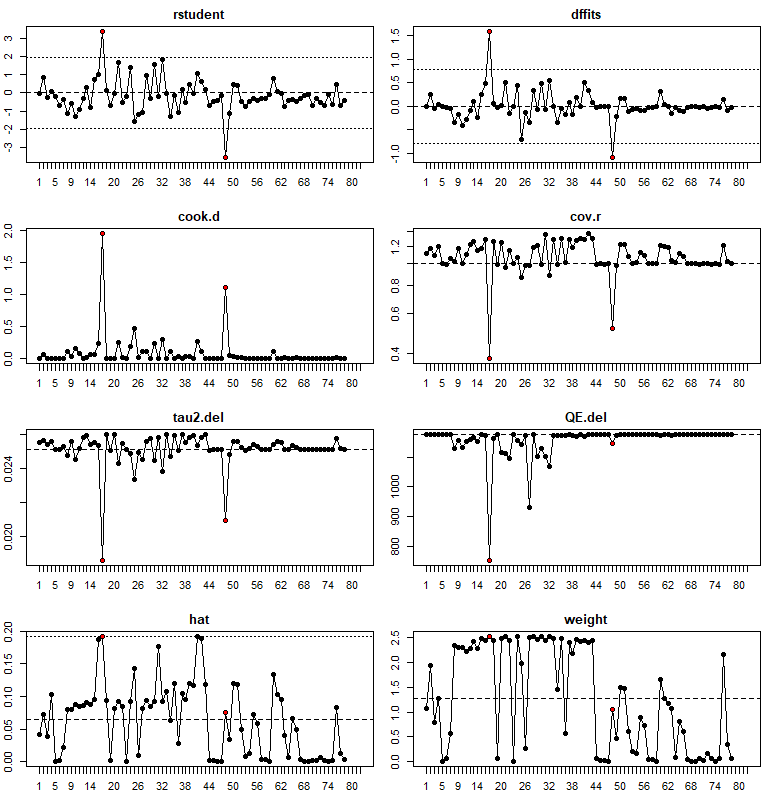


**Figure S5:** Forest plot of studies covering biosecurity, vaccination and cattle introduction. The column on the right refers to the percentage difference of the production losses before and after mitigation measures with the corresponding confidence intervals (shown in brackets). A positive percentage value indicated a benefit and negative value shows additional losses due to implemented mitigation measures. The different single letters attached before the authors’ names (left column) represent the following classification, i.e. Y=Yes; N=No and the numbers show the proportion of introduced cattle compared to the original herd size, i.e., 0.25 indicated that 25% of the cattle were replaced compared to original herd size (see Table 1). The order of the letters follows the assignment biosecurity, vaccination and cattle introduction (see also Table S2). The grey diamonds represent the effect size adjusted for the biosecurity, vaccination and cattle introduction. N.B. Full references of authors shown in the forest plots are available in the Supplementary Table S4.

**Figure S6:** Forest plot of studies covering vaccination, biosecurity and contact with (neighbouring) cattle herds. The column on the right refers to the percentage difference of the production losses before and after mitigation measures with the corresponding confidence intervals (shown in brackets). A positive percentage value indicated a benefit and negative value shows additional losses due to implemented mitigation measures. The different single letters attached before the authors’ names (left column) represent the following classification, i.e. Y=Yes; N=No. The order of the letters follows the assignment vaccination, biosecurity and contact with (neighbouring) cattle herds (see also Table S2). The grey diamonds represent the effect size adjusted for vaccination, biosecurity and contact with (neighbouring) cattle herds. N.B. Full references of authors shown in the forest plots are available in the Supplementary Table S4.

**Table S1:** Data used for the meta-analysis of the trial 1. New built data originating from studies are highlighted in light grey.

| **First Author of the Study** | **Study type** | **Study level** | **Publication year** | **Duration (years)** | **Country** | **Discount rate** | **Production system** | **Number of herds** | **Average herd size** | **Replacement rate**† | **Management system** | **In-calf cow purchase** | **Circulation rate** | **Biosecurity break** | **Virus circulation at t0** | **Prevalence (at animal level)** | **Prevalence (at herd level)** |
| --- | --- | --- | --- | --- | --- | --- | --- | --- | --- | --- | --- | --- | --- | --- | --- | --- | --- |
| Smith 2014 | Modelling | Farm | 2014 | 10 | USA | NA | Beef | 1 | 400 | 0,15 | Open | Yes | Certainty | NA | Persistent | 0,0025 | 0,1000 |
| Smith 2014 | Modelling | Farm | 2014 | 10 | USA | NA | Beef | 1 | 400 | 0,15 | Open | No | Certainty | NA | Persistent | 0,0025 | 0,1000 |
| Smith 2014 | Modelling | Farm | 2014 | 10 | USA | NA | Beef | 1 | 400 | 0,15 | Open | Yes | Certainty | NA | Persistent | 0,0025 | 0,1000 |
| Smith 2014 | Modelling | Farm | 2014 | 10 | USA | NA | Beef | 1 | 400 | 0,15 | Open | No | Certainty | NA | Persistent | 0,0025 | 0,1000 |
| Smith 2014 | Modelling | Farm | 2014 | 10 | USA | NA | Beef | 1 | 100 | 0,15 | Open | Yes | Certainty | NA | Persistent | 0,0100 | 0,1000 |
| Smith 2014 | Modelling | Farm | 2014 | 10 | USA | NA | Beef | 1 | 100 | 0,15 | Open | No | Certainty | NA | Persistent | 0,0100 | 0,1000 |
| Smith 2014 | Modelling | Farm | 2014 | 10 | USA | NA | Beef | 1 | 100 | 0,15 | Open | Yes | Certainty | NA | Persistent | 0,0100 | 0,1000 |
| Smith 2014 | Modelling | Farm | 2014 | 10 | USA | NA | Beef | 1 | 100 | 0,15 | Open | No | Certainty | NA | Persistent | 0,0100 | 0,1000 |
| Smith 2014 | Modelling | Farm | 2014 | 10 | USA | NA | Beef | 1 | 50 | 0,15 | Open | Yes | Certainty | NA | Persistent | 0,0200 | 0,1000 |
| Smith 2014 | Modelling | Farm | 2014 | 10 | USA | NA | Beef | 1 | 50 | 0,15 | Open | No | Certainty | NA | Persistent | 0,0200 | 0,1000 |
| Smith 2014 | Modelling | Farm | 2014 | 10 | USA | NA | Beef | 1 | 50 | 0,15 | Open | Yes | Certainty | NA | Persistent | 0,0200 | 0,1000 |
| Smith 2014 | Modelling | Farm | 2014 | 10 | USA | NA | Beef | 1 | 50 | 0,15 | Open | No | Certainty | NA | Persistent | 0,0200 | 0,1000 |
| Larson 2002 | Descriptive | Farm | 2002 | 10 | USA | NA | Beef | 1 | NA | 0,15 | Open | NA | Certainty | NA | Persistent | 0,0100 | 0,0100 |
| Larson 2002 | Descriptive | Farm | 2002 | 10 | USA | NA | Beef | 1 | NA | 0,15 | Open | NA | Certainty | NA | Persistent | 0,1500 | 0,1500 |
| Pasman 1994 | Modelling | Farm | 1994 | 1 | Netherland | NA | Dairy | 1 | 100 | NA | Open | NA | Certainty | NA | Persistent | NA | NA |
| Reichel 2008 | Modelling | Farm | 2008 | 10 | New Zealand | 0,12 | Dairy | 1 | 322 | 0,25 | Closed | NA | Certainty | NA | Transient | 0,0100 | NA |
| Reichel 2008 | Modelling | Farm | 2008 | 10 | New Zealand | 0,12 | Dairy | 1 | 322 | 0,25 | Closed | NA | Certainty | NA | Persistent | 0,0100 | NA |
| Reichel 2008 | Modelling | Farm | 2008 | 10 | New Zealand | 0,12 | Dairy | 1 | 322 | 0,25 | Closed | NA | Certainty | NA | Transient | 0,0100 | NA |
| Chi 2002 | Modelling | Farm | 2002 | 1 | Canada | NA | Dairy | 1 | 50 | NA | Open | NA | Likelihood | NA | NA | NA | NA |
| Chi 2002 | Modelling | Farm | 2002 | 1 | Canada | NA | Dairy | 1 | 50 | NA | Open | NA | Likelihood | NA | NA | NA | NA |
| Stott 2003 | Modelling | Farm | 2003 | 10 | Scotland | 0,05 | Beef | 1 | 100 | 0,15 | Closed | NA | Likelihood | Persistent | Persistent | NA | 0,5000 |
| Stott 2003 | Modelling | Farm | 2003 | 10 | Scotland | 0,05 | Beef | 1 | 100 | 0,15 | Closed | NA | Likelihood | Persistent | Persistent | NA | 0,5000 |
| Stott 2003 | Modelling | Farm | 2003 | 10 | Scotland | 0,05 | Beef | 1 | 100 | 0,15 | Closed | NA | Likelihood | Persistent | Persistent | NA | 0,5000 |
| Stott 2003 | Modelling | Farm | 2003 | 10 | Scotland | 0,05 | Beef | 1 | 100 | 0,15 | Closed | NA | Likelihood | Persistent | Persistent | NA | 0,5000 |
| Stott 2003 | Modelling | Farm | 2003 | 10 | Scotland | 0,05 | Beef | 1 | 100 | 0,15 | Closed | NA | Likelihood | Persistent | Persistent | NA | 0,5000 |
| Stott 2008 | Modelling | Farm | 2008 | 10 | Scotland | NA | Beef | 1 | 50 | Adj (average 0.15) | Closed | NA | Likelihood | Persistent | Transient | NA | 0,5000 |
| Stott 2008 | Modelling | Farm | 2008 | 10 | Scotland | NA | Beef | 1 | 50 | Adj (average 0.15) | Closed | NA | Likelihood | Persistent | Transient | NA | 0,5000 |
| Stott 2008 | Modelling | Farm | 2008 | 10 | Scotland | NA | Beef | 1 | 120 | Adj (average 0.15) | Closed | NA | Likelihood | Persistent | Transient | NA | 0,5000 |
| Stott 2008 | Modelling | Farm | 2008 | 10 | Scotland | NA | Beef | 1 | 120 | Adj (average 0.15) | Closed | NA | Likelihood | Persistent | Transient | NA | 0,5000 |
| Stott 2008 | Modelling | Farm | 2008 | 10 | Scotland | NA | Beef | 1 | 50 | Adj (average 0.15) | Closed | NA | Likelihood | Persistent | Transient | NA | NA |
| Stott 2008 | Modelling | Farm | 2008 | 10 | Scotland | NA | Beef | 1 | 50 | Adj (average 0.15) | Closed | NA | Likelihood | Persistent | Transient | NA | NA |
| Stott 2008 | Modelling | Farm | 2008 | 10 | Scotland | NA | Beef | 1 | 50 | Adj (average 0.15) | Closed | NA | Likelihood | Persistent | Transient | NA | NA |
| Stott 2008 | Modelling | Farm | 2008 | 10 | Scotland | NA | Beef | 1 | 50 | Adj (average 0.15) | Closed | NA | Likelihood | Persistent | Transient | NA | NA |
| Stott 2008 | Modelling | Farm | 2008 | 10 | Scotland | NA | Beef | 1 | 50 | Adj (average 0.15) | Closed | NA | Likelihood | Persistent | Transient | NA | NA |
| Stott 2008 | Modelling | Farm | 2008 | 10 | Scotland | NA | Beef | 1 | 50 | Adj (average 0.15) | Closed | NA | Likelihood | Persistent | Transient | NA | NA |
| Stott 2008 | Modelling | Farm | 2008 | 10 | Scotland | NA | Beef | 1 | 50 | Adj (average 0.15) | Closed | NA | Likelihood | Persistent | Transient | NA | NA |
| Stott 2008 | Modelling | Farm | 2008 | 10 | Scotland | NA | Beef | 1 | 50 | Adj (average 0.15) | Closed | NA | Likelihood | Persistent | Transient | NA | NA |
| Stott 2008 | Modelling | Farm | 2008 | 10 | Scotland | NA | Beef | 1 | 50 | Adj (average 0.15) | Closed | NA | Likelihood | Persistent | Transient | NA | NA |
| Stott 2008 | Modelling | Farm | 2008 | 10 | Scotland | NA | Beef | 1 | 50 | Adj (average 0.15) | Closed | NA | Likelihood | Persistent | Transient | NA | NA |
| Stott 2008 | Modelling | Farm | 2008 | 10 | Scotland | NA | Beef | 1 | 120 | Adj (average 0.15) | Closed | NA | Likelihood | Persistent | Transient | NA | NA |
| Stott 2008 | Modelling | Farm | 2008 | 10 | Scotland | NA | Beef | 1 | 120 | Adj (average 0.15) | Closed | NA | Likelihood | Persistent | Transient | NA | NA |
| Stott 2008 | Modelling | Farm | 2008 | 10 | Scotland | NA | Beef | 1 | 120 | Adj (average 0.15) | Closed | NA | Likelihood | Persistent | Transient | NA | NA |
| Stott 2008 | Modelling | Farm | 2008 | 10 | Scotland | NA | Beef | 1 | 120 | Adj (average 0.15) | Closed | NA | Likelihood | Persistent | Transient | NA | NA |
| Stott 2008 | Modelling | Farm | 2008 | 10 | Scotland | NA | Beef | 1 | 120 | Adj (average 0.15) | Closed | NA | Likelihood | Persistent | Transient | NA | NA |
| Stott 2008 | Modelling | Farm | 2008 | 10 | Scotland | NA | Beef | 1 | 120 | Adj (average 0.15) | Closed | NA | Likelihood | Persistent | Transient | NA | NA |
| Stott 2008 | Modelling | Farm | 2008 | 10 | Scotland | NA | Beef | 1 | 120 | Adj (average 0.15) | Closed | NA | Likelihood | Persistent | Transient | NA | NA |
| Stott 2008 | Modelling | Farm | 2008 | 10 | Scotland | NA | Beef | 1 | 120 | Adj (average 0.15) | Closed | NA | Likelihood | Persistent | Transient | NA | NA |
| Stott 2008 | Modelling | Farm | 2008 | 10 | Scotland | NA | Beef | 1 | 120 | Adj (average 0.15) | Closed | NA | Likelihood | Persistent | Transient | NA | NA |
| Stott 2008 | Modelling | Farm | 2008 | 10 | Scotland | NA | Beef | 1 | 120 | Adj (average 0.15) | Closed | NA | Likelihood | Persistent | Transient | NA | NA |
| Stott 2010 | Modelling | Farm | 2010 | 10 | Scotland | 0,05 | Beef | 1 | 60 | 0,15 | Open | NA | Likelihood | No | Persistent | 0,0052 | 0,1016 |
| Stott 2010 | Modelling | Farm | 2010 | 10 | Scotland | 0,05 | Beef | 1 | 60 | 0,15 | Open | NA | Likelihood | Transient | Persistent | 0,0052 | 0,1016 |
| Stott 2010 | Modelling | Farm | 2010 | 10 | Scotland | 0,05 | Beef | 1 | 60 | 0,15 | Open | NA | Likelihood | Transient | Persistent | 0,0052 | 0,1016 |
| Stott 2010 | Modelling | Farm | 2010 | 10 | Scotland | 0,05 | Beef | 1 | 60 | 0,15 | Open | NA | Likelihood | Transient | Persistent | 0,0052 | 0,1016 |
| Stott 2010 | Modelling | Farm | 2010 | 10 | Scotland | 0,05 | Beef | 1 | 60 | 0,15 | Open | NA | Likelihood | No | Persistent | 0,0052 | 0,1016 |
| Stott 2010 | Modelling | Farm | 2010 | 10 | Scotland | 0,05 | Beef | 1 | 60 | 0,15 | Open | NA | Likelihood | Transient | Persistent | 0,0052 | 0,1016 |
| Stott 2010 | Modelling | Farm | 2010 | 10 | Scotland | 0,05 | Beef | 1 | 60 | 0,15 | Open | NA | Likelihood | Transient | Persistent | 0,0052 | 0,1016 |
| Stott 2010 | Modelling | Farm | 2010 | 10 | Scotland | 0,05 | Beef | 1 | 60 | 0,15 | Open | NA | Likelihood | Transient | Persistent | 0,0052 | 0,1016 |
| Stott 2010 | Modelling | Farm | 2010 | 10 | Scotland | 0,05 | Beef | 1 | 60 | 0,15 | Open | NA | Likelihood | Transient | Transient | 0,0052 | 0,1016 |
| Stott 2010 | Modelling | Farm | 2010 | 10 | Scotland | 0,05 | Beef | 1 | 60 | 0,15 | Open | NA | Likelihood | Transient | Transient | 0,0052 | 0,1016 |
| Stott 2010 | Modelling | Farm | 2010 | 10 | Scotland | 0,05 | Beef | 1 | 60 | 0,15 | Open | NA | Likelihood | Transient | Transient | 0,0052 | 0,1016 |
| Stott 2010 | Modelling | Farm | 2010 | 10 | Scotland | 0,05 | Beef | 1 | 60 | 0,15 | Open | NA | Likelihood | Persistent | Transient | 0,0052 | 0,1016 |
| Stott 2010 | Modelling | Farm | 2010 | 10 | Scotland | 0,05 | Beef | 1 | 60 | 0,15 | Open | NA | Likelihood | Persistent | Transient | 0,0052 | 0,1016 |
| Gunn 2004 | Modelling | Farm | 2004 | 10 | Scotland | 0,05 | Beef | 1 | 230 | 0,15 | Closed | No | Likelihood | NA | Persistent | NA | NA |
| Gunn 2004 | Modelling | Farm | 2004 | 10 | Scotland | 0,05 | Beef | 1 | 230 | 0,15 | Closed | No | Likelihood | NA | Persistent | NA | NA |
| Gunn 2004 | Modelling | Farm | 2004 | 10 | Scotland | 0,05 | Beef | 1 | 230 | 0,15 | Closed | No | Likelihood | NA | Persistent | NA | NA |
| Valle 2005 | Modelling | National | 2005 | 10 | Norway | 0,04 | Mixed | 27800 | 35 | NA | Open | NA | Likelihood | NA | NA | 0,0090 | NA |
| Stott 2012 | Modelling | National | 2012 | 6 | Ireland | 0,05 | Beef | 63770 | 14,2 | 0,15 | Open | NA | Likelihood | NA | NA | 0,0075 | 0,2500 |
| Stott 2012 | Modelling | National | 2012 | 6 | Ireland | 0,05 | Dairy | 24267 | 47 | 0,15 | Open | NA | Likelihood | NA | NA | 0,0075 | 0,2500 |
| Stott 2012 | Modelling | National | 2012 | 6 | Ireland | 0,05 | Dairy | 24267 | 47 | 0,15 | Open | NA | Likelihood | NA | NA | 0,0075 | 0,2500 |
| Stott 2012 | Modelling | National | 2012 | 6 | Ireland | 0,05 | Dairy | 24267 | 47 | 0,15 | Open | NA | Likelihood | NA | Persistent | 0,0075 | 0,2500 |
| Thomann 2017 | Modelling | National | 2017 | 13 | Switzerland | 0,02 | Dairy | 2804 | 15 | 0,28 | Open | NA | Likelihood | NA | NA | 0,0080 | 0,2000 |
| Thomann 2017 | Modelling | National | 2017 | 13 | Switzerland | 0,02 | Dairy | 1682 | 20 | 0,28 | Open | NA | Likelihood | NA | NA | 0,0080 | 0,2000 |
| Thomann 2017 | Modelling | National | 2017 | 13 | Switzerland | 0,02 | Dairy | 1122 | 30 | 0,28 | Open | NA | Likelihood | NA | NA | 0,0080 | 0,2000 |
| Häsler 2012 | Modelling | National | 2012 | 10 | Switzerland | 0,03 | Mixed | 43267 | NA (1.52 mill.) | NA | Open | NA | Likelihood | NA | NA | 0,0080 | NA |
| Häsler 2012 | Modelling | National | 2012 | 10 | Switzerland | 0,03 | Mixed | 43267 | NA (1.48 mill.) | NA | Open | NA | Likelihood | NA | NA | 0,0080 | NA |
| Bennett 1986 | Descriptive | National | 1986 | 5 | United Kingdom | NA | Mixed | NA | NA | NA | Open | NA | Likelihood | NA | NA | NA | NA |
| Bitsch 1994 | Descriptive | National | 1994 | 3 | Denmark | NA | Mixed | 25000 | 38 | NA | Open | NA | Likelihood | NA | NA | NA | NA |
| Dufour 1999 | Modelling | National | 1999 | 20 | France | NA | Mixed | 3300 | 70 | NA | Open | NA | Likelihood | NA | NA | 0,0090 | NA |
| Weldegebriel 2009 | Modelling | Regional | 2009 | 10 | Scotland | 0,05 | Dairy | 1874 | 106 | 0,3 | Closed | No | Likelihood | NA | Persistent | 0,0500 | 0,0053 |
| Marschik 2018 | Descriptive | Regional | 2018 | 18 | Austria | NA | Mixed | 16269 | 20 | NA | Open | NA | Certainty | NA | Persistent | 0,0050 | 0,0800 |

† Adj=Adjusted replacement rate during simulation runs

**Table S1:** Continued

| **First Author of the Study** | **BVDV status at the beginning** | **Spread** | **Transmission rate** | **Contact with neighbouring cattle herds** | **BVDV introduction risk (1= Low; 2=Moderate; 3=High)** | **BVDV initial prevalence (1= Low; 2=Moderate; 3=High)** | **BVDV circulation intensity (1= Low; 2=Moderate; 3=High)** | **BVDV circulation duration (1= Low; 2=Moderate; 3=High)** | **BVDV production losses (mean/annual/animal) in Euro 2018** |
| --- | --- | --- | --- | --- | --- | --- | --- | --- | --- |
| Smith 2014 | Free/Susceptible | Epidemic/Endemic | High | Yes | 3 | 3 | 2 | 1 | 55,33 |
| Smith 2014 | Free/Susceptible | Epidemic/Endemic | Moderate | No | 3 | 3 | 1 | 2 | 56,59 |
| Smith 2014 | Free/Susceptible | Epidemic/Endemic | High | Yes | 3 | 3 | 2 | 1 | 87,42 |
| Smith 2014 | Free/Susceptible | Epidemic/Endemic | Moderate | No | 2 | 3 | 1 | 2 | 49,83 |
| Smith 2014 | Free/Susceptible | Epidemic/Endemic | High | Yes | 3 | 3 | 2 | 1 | 47,30 |
| Smith 2014 | Free/Susceptible | Epidemic/Endemic | Moderate | No | 3 | 3 | 1 | 2 | 35,47 |
| Smith 2014 | Free/Susceptible | Epidemic/Endemic | High | Yes | 3 | 3 | 2 | 1 | 43,92 |
| Smith 2014 | Free/Susceptible | Epidemic/Endemic | Moderate | No | 2 | 3 | 1 | 2 | 25,34 |
| Smith 2014 | Free/Susceptible | Epidemic/Endemic | High | Yes | 3 | 3 | 2 | 1 | 30,41 |
| Smith 2014 | Free/Susceptible | Epidemic/Endemic | Moderate | No | 3 | 3 | 1 | 2 | 20,27 |
| Smith 2014 | Free/Susceptible | Epidemic/Endemic | High | Yes | 3 | 3 | 2 | 1 | 23,65 |
| Smith 2014 | Free/Susceptible | Epidemic/Endemic | Moderate | No | 2 | 3 | 1 | 2 | 0,00 |
| Larson 2002 | Free/Susceptible | Endemic | Moderate | NA | 1 | 2 | 1 | 2 | 32,37 |
| Larson 2002 | Free/Susceptible | Endemic | Moderate | NA | 1 | 2 | 1 | 2 | 42,93 |
| Pasman 1994 | Free/Susceptible | Epidemic | High | NA | 3 | 3 | 3 | 3 | 73,11 |
| Reichel 2008 | Free/Susceptible | Epidemic | High | No | 3 | 3 | 1 | 1 | 2,81 |
| Reichel 2008 | Free/Susceptible | Endemic | Low | No | 3 | 2 | 1 | 1 | 17,78 |
| Reichel 2008 | Free/Susceptible | Epidemic | High | No | 3 | 3 | 3 | 1 | 44,19 |
| Chi 2002 | Infected (Susceptible, Transient, Immune, Persistent) | Endemic | Low | No | 2 | 2 | 1 | 2 | 42,33 |
| Chi 2002 | Infected (Susceptible, Transient, Immune, Persistent) | Endemic | Low | No | 3 | 3 | 2 | 2 | 63,19 |
| Stott 2003 | Free/Susceptible | Epidemic | High | No | 3 | 3 | 2 | 1 | 40,18 |
| Stott 2003 | Free/Susceptible | Epidemic | High | No | 2 | 3 | 2 | 1 | 31,38 |
| Stott 2003 | Free/Susceptible | Epidemic | Moderate | No | 1 | 3 | 2 | 1 | 25,22 |
| Stott 2003 | Infected (Susceptible, Transient, Immune, Persistent) | Epidemic/Endemic | Moderate | No | 3 | 2 | 1 | 1 | 29,21 |
| Stott 2003 | Infected (Susceptible, Transient, Immune, Persistent) | Epidemic/Endemic | Moderate | No | 2 | 2 | 1 | 1 | 27,86 |
| Stott 2008 | Free/Susceptible | Epidemic | High | No | 3 | 3 | 2 | 1 | 71,44 |
| Stott 2008 | Infected (Susceptible, Transient, Immune, Persistent) | Endemic | Moderate | No | 3 | 1 | 1 | 1 | 52,08 |
| Stott 2008 | Free/Susceptible | Epidemic | High | No | 3 | 3 | 2 | 1 | 70,31 |
| Stott 2008 | Infected (Susceptible, Transient, Immune, Persistent) | Endemic | Moderate | No | 3 | 2 | 1 | 1 | 45,64 |
| Stott 2008 | Free/Susceptible | Epidemic | High | No | 1 | 1 | 2 | 2 | 23,73 |
| Stott 2008 | Free/Susceptible | Epidemic | High | No | 1 | 2 | 2 | 2 | 24,06 |
| Stott 2008 | Free/Susceptible | Epidemic | High | No | 1 | 2 | 2 | 2 | 40,34 |
| Stott 2008 | Free/Susceptible | Epidemic | High | No | 2 | 3 | 2 | 1 | 50,80 |
| Stott 2008 | Free/Susceptible | Epidemic | High | No | 3 | 3 | 2 | 3 | 49,31 |
| Stott 2008 | Infected (Susceptible, Transient, Immune, Persistent) | Endemic | Moderate | No | 1 | 1 | 1 | 2 | 27,94 |
| Stott 2008 | Infected (Susceptible, Transient, Immune, Persistent) | Endemic | Moderate | No | 1 | 2 | 1 | 2 | 25,80 |
| Stott 2008 | Infected (Susceptible, Transient, Immune, Persistent) | Endemic | Moderate | No | 1 | 2 | 1 | 3 | 41,24 |
| Stott 2008 | Infected (Susceptible, Transient, Immune, Persistent) | Endemic | Moderate | No | 2 | 3 | 1 | 2 | 41,50 |
| Stott 2008 | Infected (Susceptible, Transient, Immune, Persistent) | Endemic | Moderate | No | 3 | 3 | 1 | 1 | 48,05 |
| Stott 2008 | Infected (Susceptible, Transient, Immune, Persistent) | Epidemic | High | No | 1 | 1 | 2 | 2 | 18,90 |
| Stott 2008 | Infected (Susceptible, Transient, Immune, Persistent) | Epidemic | High | No | 1 | 2 | 2 | 2 | 28,01 |
| Stott 2008 | Infected (Susceptible, Transient, Immune, Persistent) | Epidemic | High | No | 1 | 2 | 2 | 2 | 29,80 |
| Stott 2008 | Infected (Susceptible, Transient, Immune, Persistent) | Epidemic | High | No | 2 | 3 | 2 | 2 | 53,00 |
| Stott 2008 | Infected (Susceptible, Transient, Immune, Persistent) | Epidemic | High | No | 3 | 3 | 2 | 1 | 31,83 |
| Stott 2008 | Infected (Susceptible, Transient, Immune, Persistent) | Endemic | Moderate | No | 1 | 1 | 1 | 2 | 23,47 |
| Stott 2008 | Infected (Susceptible, Transient, Immune, Persistent) | Endemic | Moderate | No | 1 | 2 | 1 | 2 | 25,74 |
| Stott 2008 | Infected (Susceptible, Transient, Immune, Persistent) | Endemic | Moderate | No | 1 | 2 | 1 | 2 | 36,04 |
| Stott 2008 | Infected (Susceptible, Transient, Immune, Persistent) | Endemic | Moderate | No | 2 | 3 | 1 | 3 | 41,13 |
| Stott 2008 | Infected (Susceptible, Transient, Immune, Persistent) | Endemic | Moderate | No | 3 | 3 | 1 | 1 | 41,01 |
| Stott 2010 | Infected (Susceptible, Transient, Immune, Persistent) | Epidemic | High | No | 2 | 2 | 1 | 1 | 40,51 |
| Stott 2010 | Infected (Susceptible, Transient, Immune, Persistent) | Epidemic | High | Yes | 2 | 2 | 1 | 1 | 40,84 |
| Stott 2010 | Infected (Susceptible, Transient, Immune, Persistent) | Epidemic | High | Yes | 2 | 2 | 1 | 1 | 47,70 |
| Stott 2010 | Infected (Susceptible, Transient, Immune, Persistent) | Epidemic | High | Yes | 2 | 2 | 1 | 2 | 50,64 |
| Stott 2010 | Infected (Susceptible, Transient, Immune, Persistent) | Epidemic/Endemic | High | No | 3 | 3 | 2 | 1 | 47,70 |
| Stott 2010 | Infected (Susceptible, Transient, Immune, Persistent) | Epidemic/Endemic | High | No | 3 | 3 | 2 | 2 | 47,70 |
| Stott 2010 | Infected (Susceptible, Transient, Immune, Persistent) | Epidemic/Endemic | High | No | 3 | 3 | 2 | 2 | 49,66 |
| Stott 2010 | Infected (Susceptible, Transient, Immune, Persistent) | Epidemic/Endemic | High | No | 3 | 3 | 1 | 2 | 55,87 |
| Stott 2010 | Infected (Susceptible, Transient, Immune, Persistent) | Epidemic/Endemic | High | No | 2 | 3 | 1 | 1 | 32,02 |
| Stott 2010 | Infected (Susceptible, Transient, Immune, Persistent) | Epidemic/Endemic | High | No | 2 | 3 | 2 | 2 | 43,13 |
| Stott 2010 | Infected (Susceptible, Transient, Immune, Persistent) | Epidemic/Endemic | High | No | 2 | 3 | 2 | 2 | 48,35 |
| Stott 2010 | Infected (Susceptible, Transient, Immune, Persistent) | Epidemic/Endemic | High | No | 3 | 3 | 1 | 1 | 23,52 |
| Stott 2010 | Infected (Susceptible, Transient, Immune, Persistent) | Epidemic/Endemic | High | No | 3 | 3 | 1 | 1 | 3,27 |
| Gunn 2004 | Infected (Susceptible, Transient, Immune, Persistent) | Epidemic | Low | NA | 2 | 3 | 1 | 1 | 73,01 |
| Gunn 2004 | Infected (Susceptible, Transient, Immune, Persistent) | Epidemic | Moderate | NA | 2 | 3 | 2 | 2 | 77,82 |
| Gunn 2004 | Infected (Susceptible, Transient, Immune, Persistent) | Epidemic | High | NA | 2 | 3 | 2 | 3 | 80,12 |
| Valle 2005 | Infected (Susceptible, Transient, Immune, Persistent) | Endemic | Moderate | NA | 1 | 1 | 1 | 3 | 16,10 |
| Stott 2012 | Infected (Susceptible, Transient, Immune, Persistent) | Endemic | Moderate | No | 1 | 2 | 1 | 3 | 37,14 |
| Stott 2012 | Infected (Susceptible, Transient, Immune, Persistent) | Endemic | Moderate | No | 1 | 2 | 1 | 3 | 73,12 |
| Stott 2012 | Free/Susceptible | Endemic | High | No | 1 | 2 | 3 | 3 | 66,15 |
| Stott 2012 | Infected (Susceptible, Transient, Immune, Persistent) | Endemic | Moderate | No | 1 | 2 | 3 | 3 | 80,08 |
| Thomann 2017 | Infected (Susceptible, Transient, Immune, Persistent) | Endemic | High | NA | 1 | 2 | 1 | 3 | 70,33 |
| Thomann 2017 | Infected (Susceptible, Transient, Immune, Persistent) | Endemic | Moderate | NA | 1 | 2 | 1 | 3 | 69,54 |
| Thomann 2017 | Infected (Susceptible, Transient, Immune, Persistent) | Endemic | Low | NA | 1 | 2 | 1 | 3 | 67,17 |
| Häsler 2012 | Infected (Susceptible, Transient, Immune, Persistent) | Endemic | Low | NA | 1 | 2 | 1 | 3 | 8,33 |
| Häsler 2012 | Infected (Susceptible, Transient, Immune, Persistent) | Endemic | Low | NA | 1 | 2 | 1 | 3 | 7,66 |
| Bennett 1986 | Infected (Susceptible, Transient, Immune, Persistent) | Endemic | High | NA | 1 | 2 | 1 | 3 | 10,82 |
| Bitsch 1994 | Infected (Susceptible, Transient, Immune, Persistent) | Endemic | High | NA | 1 | 3 | 1 | 3 | 17,07 |
| Dufour 1999 | Infected (Susceptible, Transient, Immune, Persistent) | Endemic | Low | NA | 1 | 2 | 1 | 3 | 16,27 |
| Weldegebriel 2009 | Free/Susceptible | Endemic | Moderate | NA | 1 | 2 | 1 | 3 | 32,90 |
| Marschik 2018 | Infected (Susceptible, Transient, Immune, Persistent) | Endemic | Moderate | NA | 1 | 2 | 1 | 3 | 3,73 |

**Table S2:** Data used for the meta-analysis of the trial 2. New built data originating from studies are highlighted in light grey.

| **First Author of the Study** | **Study type** | **Study level** | **Publication year** | **Duration (years)** | **Country** | **Discount rate** | **Production system** | **Average herd size** | **Replacement rate** | **Management system** | **Circulation rate** | **BVDV status at the beginning** | **Spread** | **Transmission rate** |
| --- | --- | --- | --- | --- | --- | --- | --- | --- | --- | --- | --- | --- | --- | --- |
| Reichel 2008 | Modelling | Farm | 2008 | 10 | New Zealand | 0,12 | Dairy | 322 | 0,25 | Closed | Certainty | Free/Susceptible | Endemic | Low |
| Reichel 2008 | Modelling | Farm | 2008 | 10 | New Zealand | 0,12 | Dairy | 322 | 0,25 | Closed | Certainty | Free/Susceptible | Endemic | Low |
| Reichel 2008 | Modelling | Farm | 2008 | 10 | New Zealand | 0,12 | Dairy | 322 | 0,25 | Closed | Certainty | Free/Susceptible | Endemic | Low |
| Reichel 2008 | Modelling | Farm | 2008 | 10 | New Zealand | 0,12 | Dairy | 322 | 0,25 | Closed | Certainty | Free/Susceptible | Endemic | Low |
| Reichel 2008 | Modelling | Farm | 2008 | 10 | New Zealand | 0,12 | Dairy | 322 | 0,25 | Closed | Certainty | Free/Susceptible | Endemic | Low |
| Reichel 2008 | Modelling | Farm | 2008 | 10 | New Zealand | 0,12 | Dairy | 322 | 0,25 | Closed | Certainty | Free/Susceptible | Endemic | Low |
| Reichel 2008 | Modelling | Farm | 2008 | 10 | New Zealand | 0,12 | Dairy | 322 | 0,25 | Closed | Certainty | Free/Susceptible | Endemic | Low |
| Smith 2014 | Modelling | Farm | 2014 | 10 | USA | NA | Beef | 400 | 0,15 | Open | Certainty | Free/Susceptible | Epidemic/Endemic | High |
| Smith 2014 | Modelling | Farm | 2014 | 10 | USA | NA | Beef | 400 | 0,15 | Open | Certainty | Free/Susceptible | Epidemic/Endemic | Moderate |
| Smith 2014 | Modelling | Farm | 2014 | 10 | USA | NA | Beef | 400 | 0,15 | Open | Certainty | Free/Susceptible | Epidemic/Endemic | High |
| Smith 2014 | Modelling | Farm | 2014 | 10 | USA | NA | Beef | 400 | 0,15 | Open | Certainty | Free/Susceptible | Epidemic/Endemic | Moderate |
| Smith 2014 | Modelling | Farm | 2014 | 10 | USA | NA | Beef | 100 | 0,15 | Open | Certainty | Free/Susceptible | Epidemic/Endemic | High |
| Smith 2014 | Modelling | Farm | 2014 | 10 | USA | NA | Beef | 100 | 0,15 | Open | Certainty | Free/Susceptible | Epidemic/Endemic | Moderate |
| Smith 2014 | Modelling | Farm | 2014 | 10 | USA | NA | Beef | 100 | 0,15 | Open | Certainty | Free/Susceptible | Epidemic/Endemic | High |
| Smith 2014 | Modelling | Farm | 2014 | 10 | USA | NA | Beef | 100 | 0,15 | Open | Certainty | Free/Susceptible | Epidemic/Endemic | Moderate |
| Smith 2014 | Modelling | Farm | 2014 | 10 | USA | NA | Beef | 50 | 0,15 | Open | Certainty | Free/Susceptible | Epidemic/Endemic | High |
| Smith 2014 | Modelling | Farm | 2014 | 10 | USA | NA | Beef | 50 | 0,15 | Open | Certainty | Free/Susceptible | Epidemic/Endemic | Moderate |
| Smith 2014 | Modelling | Farm | 2014 | 10 | USA | NA | Beef | 50 | 0,15 | Open | Certainty | Free/Susceptible | Epidemic/Endemic | High |
| Smith 2014 | Modelling | Farm | 2014 | 10 | USA | NA | Beef | 400 | 0,15 | Open | Certainty | Free/Susceptible | Epidemic/Endemic | High |
| Smith 2014 | Modelling | Farm | 2014 | 10 | USA | NA | Beef | 400 | 0,15 | Open | Certainty | Free/Susceptible | Epidemic/Endemic | Moderate |
| Smith 2014 | Modelling | Farm | 2014 | 10 | USA | NA | Beef | 400 | 0,15 | Open | Certainty | Free/Susceptible | Epidemic/Endemic | High |
| Smith 2014 | Modelling | Farm | 2014 | 10 | USA | NA | Beef | 400 | 0,15 | Open | Certainty | Free/Susceptible | Epidemic/Endemic | Moderate |
| Smith 2014 | Modelling | Farm | 2014 | 10 | USA | NA | Beef | 100 | 0,15 | Open | Certainty | Free/Susceptible | Epidemic/Endemic | High |
| Smith 2014 | Modelling | Farm | 2014 | 10 | USA | NA | Beef | 100 | 0,15 | Open | Certainty | Free/Susceptible | Epidemic/Endemic | Moderate |
| Smith 2014 | Modelling | Farm | 2014 | 10 | USA | NA | Beef | 100 | 0,15 | Open | Certainty | Free/Susceptible | Epidemic/Endemic | High |
| Smith 2014 | Modelling | Farm | 2014 | 10 | USA | NA | Beef | 100 | 0,15 | Open | Certainty | Free/Susceptible | Epidemic/Endemic | Moderate |
| Smith 2014 | Modelling | Farm | 2014 | 10 | USA | NA | Beef | 50 | 0,15 | Open | Certainty | Free/Susceptible | Epidemic/Endemic | High |
| Smith 2014 | Modelling | Farm | 2014 | 10 | USA | NA | Beef | 50 | 0,15 | Open | Certainty | Free/Susceptible | Epidemic/Endemic | Moderate |
| Smith 2014 | Modelling | Farm | 2014 | 10 | USA | NA | Beef | 50 | 0,15 | Open | Certainty | Free/Susceptible | Epidemic/Endemic | High |
| Smith 2014 | Modelling | Farm | 2014 | 10 | USA | NA | Beef | 400 | 0,15 | Open | Certainty | Free/Susceptible | Epidemic/Endemic | High |
| Smith 2014 | Modelling | Farm | 2014 | 10 | USA | NA | Beef | 400 | 0,15 | Open | Certainty | Free/Susceptible | Epidemic/Endemic | High |
| Smith 2014 | Modelling | Farm | 2014 | 10 | USA | NA | Beef | 100 | 0,15 | Open | Certainty | Free/Susceptible | Epidemic/Endemic | High |
| Smith 2014 | Modelling | Farm | 2014 | 10 | USA | NA | Beef | 100 | 0,15 | Open | Certainty | Free/Susceptible | Epidemic/Endemic | High |
| Smith 2014 | Modelling | Farm | 2014 | 10 | USA | NA | Beef | 50 | 0,15 | Open | Certainty | Free/Susceptible | Epidemic/Endemic | High |
| Smith 2014 | Modelling | Farm | 2014 | 10 | USA | NA | Beef | 50 | 0,15 | Open | Certainty | Free/Susceptible | Epidemic/Endemic | High |
| Smith 2014 | Modelling | Farm | 2014 | 10 | USA | NA | Beef | 400 | 0,15 | Open | Certainty | Free/Susceptible | Epidemic/Endemic | High |
| Smith 2014 | Modelling | Farm | 2014 | 10 | USA | NA | Beef | 400 | 0,15 | Open | Certainty | Free/Susceptible | Epidemic/Endemic | Moderate |
| Smith 2014 | Modelling | Farm | 2014 | 10 | USA | NA | Beef | 400 | 0,15 | Open | Certainty | Free/Susceptible | Epidemic/Endemic | High |
| Smith 2014 | Modelling | Farm | 2014 | 10 | USA | NA | Beef | 400 | 0,15 | Open | Certainty | Free/Susceptible | Epidemic/Endemic | Moderate |
| Smith 2014 | Modelling | Farm | 2014 | 10 | USA | NA | Beef | 100 | 0,15 | Open | Certainty | Free/Susceptible | Epidemic/Endemic | High |
| Smith 2014 | Modelling | Farm | 2014 | 10 | USA | NA | Beef | 100 | 0,15 | Open | Certainty | Free/Susceptible | Epidemic/Endemic | Moderate |
| Smith 2014 | Modelling | Farm | 2014 | 10 | USA | NA | Beef | 100 | 0,15 | Open | Certainty | Free/Susceptible | Epidemic/Endemic | High |
| Smith 2014 | Modelling | Farm | 2014 | 10 | USA | NA | Beef | 100 | 0,15 | Open | Certainty | Free/Susceptible | Epidemic/Endemic | Moderate |
| Smith 2014 | Modelling | Farm | 2014 | 10 | USA | NA | Beef | 50 | 0,15 | Open | Certainty | Free/Susceptible | Epidemic/Endemic | High |
| Smith 2014 | Modelling | Farm | 2014 | 10 | USA | NA | Beef | 50 | 0,15 | Open | Certainty | Free/Susceptible | Epidemic/Endemic | Moderate |
| Smith 2014 | Modelling | Farm | 2014 | 10 | USA | NA | Beef | 50 | 0,15 | Open | Certainty | Free/Susceptible | Epidemic/Endemic | High |
| Smith 2014 | Modelling | Farm | 2014 | 10 | USA | NA | Beef | 400 | 0,15 | Open | Certainty | Free/Susceptible | Epidemic/Endemic | High |
| Smith 2014 | Modelling | Farm | 2014 | 10 | USA | NA | Beef | 400 | 0,15 | Open | Certainty | Free/Susceptible | Epidemic/Endemic | Moderate |
| Smith 2014 | Modelling | Farm | 2014 | 10 | USA | NA | Beef | 100 | 0,15 | Open | Certainty | Free/Susceptible | Epidemic/Endemic | High |
| Smith 2014 | Modelling | Farm | 2014 | 10 | USA | NA | Beef | 100 | 0,15 | Open | Certainty | Free/Susceptible | Epidemic/Endemic | Moderate |
| Smith 2014 | Modelling | Farm | 2014 | 10 | USA | NA | Beef | 50 | 0,15 | Open | Certainty | Free/Susceptible | Epidemic/Endemic | High |
| Smith 2014 | Modelling | Farm | 2014 | 10 | USA | NA | Beef | 50 | 0,15 | Open | Certainty | Free/Susceptible | Epidemic/Endemic | Moderate |
| Stott 2008 | Modelling | Farm | 2008 | 10 | Scotland | NA | Beef | 50 | Adj. (average 0.15) | Closed | Likelihood | Free/Susceptible | Epidemic | High |
| Stott 2008 | Modelling | Farm | 2008 | 10 | Scotland | NA | Beef | 50 | Adj. (average 0.15) | Closed | Likelihood | Free/Susceptible | Epidemic | High |
| Stott 2008 | Modelling | Farm | 2008 | 10 | Scotland | NA | Beef | 50 | Adj. (average 0.15) | Closed | Likelihood | Free/Susceptible | Epidemic | High |
| Stott 2008 | Modelling | Farm | 2008 | 10 | Scotland | NA | Beef | 50 | Adj. (average 0.15) | Closed | Likelihood | Free/Susceptible | Epidemic | High |
| Stott 2008 | Modelling | Farm | 2008 | 10 | Scotland | NA | Beef | 50 | Adj. (average 0.15) | Closed | Likelihood | Free/Susceptible | Epidemic | High |
| Stott 2008 | Modelling | Farm | 2008 | 10 | Scotland | NA | Beef | 50 | Adj. (average 0.15) | Closed | Likelihood | Infected (Susceptible, Transient, Immune, Persistent) | Endemic | Moderate |
| Stott 2008 | Modelling | Farm | 2008 | 10 | Scotland | NA | Beef | 50 | Adj. (average 0.15) | Closed | Likelihood | Infected (Susceptible, Transient, Immune, Persistent) | Endemic | Moderate |
| Stott 2008 | Modelling | Farm | 2008 | 10 | Scotland | NA | Beef | 50 | Adj. (average 0.15) | Closed | Likelihood | Infected (Susceptible, Transient, Immune, Persistent) | Endemic | Moderate |
| Stott 2008 | Modelling | Farm | 2008 | 10 | Scotland | NA | Beef | 50 | Adj. (average 0.15) | Closed | Likelihood | Infected (Susceptible, Transient, Immune, Persistent) | Endemic | Moderate |
| Stott 2008 | Modelling | Farm | 2008 | 10 | Scotland | NA | Beef | 50 | Adj. (average 0.15) | Closed | Likelihood | Infected (Susceptible, Transient, Immune, Persistent) | Endemic | Moderate |
| Stott 2008 | Modelling | Farm | 2008 | 10 | Scotland | NA | Beef | 120 | Adj. (average 0.15) | Closed | Likelihood | Free/Susceptible | Epidemic | High |
| Stott 2008 | Modelling | Farm | 2008 | 10 | Scotland | NA | Beef | 120 | Adj. (average 0.15) | Closed | Likelihood | Free/Susceptible | Epidemic | High |
| Stott 2008 | Modelling | Farm | 2008 | 10 | Scotland | NA | Beef | 120 | Adj. (average 0.15) | Closed | Likelihood | Free/Susceptible | Epidemic | High |
| Stott 2008 | Modelling | Farm | 2008 | 10 | Scotland | NA | Beef | 120 | Adj. (average 0.15) | Closed | Likelihood | Free/Susceptible | Epidemic | High |
| Stott 2008 | Modelling | Farm | 2008 | 10 | Scotland | NA | Beef | 120 | Adj. (average 0.15) | Closed | Likelihood | Free/Susceptible | Epidemic | High |
| Stott 2008 | Modelling | Farm | 2008 | 10 | Scotland | NA | Beef | 120 | Adj. (average 0.15) | Closed | Likelihood | Infected (Susceptible, Transient, Immune, Persistent) | Endemic | Moderate |
| Stott 2008 | Modelling | Farm | 2008 | 10 | Scotland | NA | Beef | 120 | Adj. (average 0.15) | Closed | Likelihood | Infected (Susceptible, Transient, Immune, Persistent) | Endemic | Moderate |
| Stott 2008 | Modelling | Farm | 2008 | 10 | Scotland | NA | Beef | 120 | Adj. (average 0.15) | Closed | Likelihood | Infected (Susceptible, Transient, Immune, Persistent) | Endemic | Moderate |
| Stott 2008 | Modelling | Farm | 2008 | 10 | Scotland | NA | Beef | 120 | Adj. (average 0.15) | Closed | Likelihood | Infected (Susceptible, Transient, Immune, Persistent) | Endemic | Moderate |
| Stott 2008 | Modelling | Farm | 2008 | 10 | Scotland | NA | Beef | 120 | Adj. (average 0.15) | Closed | Likelihood | Infected (Susceptible, Transient, Immune, Persistent) | Endemic | Moderate |
| Stott 2003 | Modelling | Farm | 2003 | 10 | Scotland | 0,05 | Beef | 100 | 0,15 | Closed | Likelihood | Free/Susceptible | Epidemic | Moderate |
| Stott 2003 | Modelling | Farm | 2003 | 10 | Scotland | 0,05 | Beef | 100 | 0,15 | Closed | Likelihood | Free/Susceptible | Epidemic | High |
| Stott 2003 | Modelling | Farm | 2003 | 10 | Scotland | 0,05 | Beef | 100 | 0,15 | Closed | Likelihood | Free/Susceptible | Epidemic | High |
| Stott 2003 | Modelling | Farm | 2003 | 10 | Scotland | 0,05 | Beef | 100 | 0,15 | Closed | Likelihood | Free/Susceptible | Epidemic | High |
| Stott 2003 | Modelling | Farm | 2003 | 10 | Scotland | 0,05 | Beef | 100 | 0,15 | Closed | Likelihood | Infected (Susceptible, Transient, Immune, Persistent) | Epidemic/Endemic | Moderate |
| Stott 2003 | Modelling | Farm | 2003 | 10 | Scotland | 0,05 | Beef | 100 | 0,15 | Closed | Likelihood | Infected (Susceptible, Transient, Immune, Persistent) | Epidemic/Endemic | High |
| Stott 2003 | Modelling | Farm | 2003 | 10 | Scotland | 0,05 | Beef | 100 | 0,15 | Closed | Likelihood | Infected (Susceptible, Transient, Immune, Persistent) | Epidemic/Endemic | High |
| Stott 2003 | Modelling | Farm | 2003 | 10 | Scotland | 0,05 | Beef | 100 | 0,15 | Closed | Likelihood | Infected (Susceptible, Transient, Immune, Persistent) | Epidemic/Endemic | High |
| Stott 2012 | Modelling | National | 2012 | 6 | Ireland | 0,05 | Beef | 14,2 | 0,15 | Open | Likelihood | Infected (Susceptible, Transient, Immune, Persistent) | Endemic | Moderate |
| Stott 2012 | Modelling | National | 2012 | 6 | Ireland | 0,05 | Dairy | 47 | 0,15 | Open | Likelihood | Infected (Susceptible, Transient, Immune, Persistent) | Endemic | Moderate |
| Stott 2012 | Modelling | National | 2012 | 6 | Ireland | 0,05 | Dairy | 47 | 0,15 | Open | Likelihood | Infected (Susceptible, Transient, Immune, Persistent) | Endemic | Moderate |
| Santman 2015 | Modelling | National | 2015 | 10 | Netherlands | 0,02 | Dairy | NA | 0,25 | Open | Likelihood | Infected (Susceptible, Transient, Immune, Persistent) | Epidemic/Endemic | Moderate |
| Santman 2015 | Modelling | National | 2015 | 10 | Netherlands | 0,02 | Dairy | NA | 0,25 | Open | Likelihood | Infected (Susceptible, Transient, Immune, Persistent) | Epidemic/Endemic | Moderate |
| Santman 2015 | Modelling | National | 2015 | 10 | Netherlands | 0,02 | Dairy | NA | 0,25 | Open | Likelihood | Infected (Susceptible, Transient, Immune, Persistent) | Epidemic/Endemic | Moderate |

**Table S2:** Continued.

| **First Author of the Study** | **Contact with neighbouring cattle herds** | **Cattle introduction (within herd, %)** | **Biosecurity** | **Biosecurity efficacy** | **Biosecurity score (1=Low; 2=Moderate; 3=High)** | **Vaccination** | **Vaccination efficacy** | **Vaccinated population** | **Vaccination frequency** | **Vaccination score (1=Low; 2=Moderate; 3=High)** | **Testing and culling** | **Testing and Culling efficacy** | **Testing and culling score (1=Low; 2=Moderate; 3=High)** |
| --- | --- | --- | --- | --- | --- | --- | --- | --- | --- | --- | --- | --- | --- |
| Reichel 2008 | No | 0 | No | NA | 1 | Yes | > 50 | Whole Herd | 2 doses first year then annually | 2 | No | NA | 1 |
| Reichel 2008 | No | 0 | Yes | 0.85 | 3 | No | NA | None | None | 1 | No | NA | 1 |
| Reichel 2008 | No | 0 | No | NA | 1 | No | NA | None | None | 1 | Yes | 0,400 | 2 |
| Reichel 2008 | No | 0 | Yes | 0.85 | 3 | Yes | > 50 | Whole Herd | 2 doses first year then annually | 2 | No | NA | 1 |
| Reichel 2008 | No | 0 | Yes | 0.85 | 3 | Yes | > 50 | Whole Herd | 2 doses first year then annually | 2 | Yes | 0,400 | 2 |
| Reichel 2008 | No | 0 | Yes | 0.85 | 3 | No | NA | None | None | 1 | Yes | 0,400 | 2 |
| Reichel 2008 | No | 0 | No | NA | 1 | Yes | > 50 | Whole Herd | 2 doses first year then annually | 2 | Yes | 0,400 | 2 |
| Smith 2014 | Yes | 25 | No | NA | 1 | Yes | < 50 | Reproductive females | Annually | 2 | No | NA | 1 |
| Smith 2014 | Yes | 25 | No | NA | 1 | Yes | < 50 | Reproductive females | Annually | 2 | No | NA | 1 |
| Smith 2014 | No | 0 | No | NA | 1 | Yes | < 50 | Reproductive females | Annually | 2 | No | NA | 1 |
| Smith 2014 | No | 0 | No | NA | 1 | Yes | < 50 | Reproductive females | Annually | 2 | No | NA | 1 |
| Smith 2014 | Yes | 100 | No | NA | 1 | Yes | < 50 | Reproductive females | Annually | 2 | No | NA | 1 |
| Smith 2014 | Yes | 100 | No | NA | 1 | Yes | < 50 | Reproductive females | Annually | 2 | No | NA | 1 |
| Smith 2014 | No | 0 | No | NA | 1 | Yes | < 50 | Reproductive females | Annually | 2 | No | NA | 1 |
| Smith 2014 | No | 0 | No | NA | 1 | Yes | < 50 | Reproductive females | Annually | 2 | No | NA | 1 |
| Smith 2014 | Yes | 200 | No | NA | 1 | Yes | < 50 | Reproductive females | Annually | 2 | No | NA | 1 |
| Smith 2014 | Yes | 200 | No | NA | 1 | Yes | < 50 | Reproductive females | Annually | 2 | No | NA | 1 |
| Smith 2014 | No | 0 | No | NA | 1 | Yes | < 50 | Reproductive females | Annually | 2 | No | NA | 1 |
| Smith 2014 | Yes | 25 | Yes | NA | 2 | No | NA | None | None | 1 | No | NA | 1 |
| Smith 2014 | Yes | 25 | Yes | NA | 2 | No | NA | None | None | 1 | No | NA | 1 |
| Smith 2014 | No | 0 | Yes | NA | 2 | No | NA | None | None | 1 | No | NA | 1 |
| Smith 2014 | No | 0 | Yes | NA | 2 | No | NA | None | None | 1 | No | NA | 1 |
| Smith 2014 | Yes | 100 | Yes | NA | 2 | No | NA | None | None | 1 | No | NA | 1 |
| Smith 2014 | Yes | 100 | Yes | NA | 2 | No | NA | None | None | 1 | No | NA | 1 |
| Smith 2014 | No | 0 | Yes | NA | 2 | No | NA | None | None | 1 | No | NA | 1 |
| Smith 2014 | No | 0 | Yes | NA | 2 | No | NA | None | None | 1 | No | NA | 1 |
| Smith 2014 | Yes | 200 | Yes | NA | 2 | No | NA | None | None | 1 | No | NA | 1 |
| Smith 2014 | Yes | 200 | Yes | NA | 2 | No | NA | None | None | 1 | No | NA | 1 |
| Smith 2014 | No | 0 | Yes | NA | 2 | No | NA | None | None | 1 | No | NA | 1 |
| Smith 2014 | Yes | 25 | Yes | NA | 2 | No | NA | None | None | 1 | No | NA | 1 |
| Smith 2014 | No | 0 | Yes | NA | 2 | No | NA | None | None | 1 | No | NA | 1 |
| Smith 2014 | Yes | 100 | Yes | NA | 2 | No | NA | None | None | 1 | No | NA | 1 |
| Smith 2014 | No | 0 | Yes | NA | 2 | No | NA | None | None | 1 | No | NA | 1 |
| Smith 2014 | Yes | 200 | Yes | NA | 2 | No | NA | None | None | 1 | No | NA | 1 |
| Smith 2014 | No | 0 | Yes | NA | 2 | No | NA | None | None | 1 | No | NA | 1 |
| Smith 2014 | Yes | 25 | No | NA | 1 | No | NA | None | None | 1 | Yes | 0,896 | 3 |
| Smith 2014 | Yes | 25 | No | NA | 1 | No | NA | None | None | 1 | Yes | 0,896 | 3 |
| Smith 2014 | No | 0 | No | NA | 1 | No | NA | None | None | 1 | Yes | 0,896 | 3 |
| Smith 2014 | No | 0 | No | NA | 1 | No | NA | None | None | 1 | Yes | 0,896 | 3 |
| Smith 2014 | Yes | 100 | No | NA | 1 | No | NA | None | None | 1 | Yes | 0,896 | 3 |
| Smith 2014 | Yes | 100 | No | NA | 1 | No | NA | None | None | 1 | Yes | 0,896 | 3 |
| Smith 2014 | No | 0 | No | NA | 1 | No | NA | None | None | 1 | Yes | 0,896 | 3 |
| Smith 2014 | No | 0 | No | NA | 1 | No | NA | None | None | 1 | Yes | 0,896 | 3 |
| Smith 2014 | Yes | 200 | No | NA | 1 | No | NA | None | None | 1 | Yes | 0,896 | 3 |
| Smith 2014 | Yes | 200 | No | NA | 1 | No | NA | None | None | 1 | Yes | 0,896 | 3 |
| Smith 2014 | No | 0 | No | NA | 1 | No | NA | None | None | 1 | Yes | 0,896 | 3 |
| Smith 2014 | Yes | 25 | Yes | NA | 2 | No | NA | None | None | 1 | No | NA | 1 |
| Smith 2014 | Yes | 25 | Yes | NA | 2 | No | NA | None | None | 1 | No | NA | 1 |
| Smith 2014 | Yes | 100 | Yes | NA | 2 | No | NA | None | None | 1 | No | NA | 1 |
| Smith 2014 | Yes | 100 | Yes | NA | 2 | No | NA | None | None | 1 | No | NA | 1 |
| Smith 2014 | Yes | 200 | Yes | NA | 2 | No | NA | None | None | 1 | No | NA | 1 |
| Smith 2014 | Yes | 200 | Yes | NA | 2 | No | NA | None | None | 1 | No | NA | 1 |
| Stott 2008 | No | 0 | Yes | 0.56 | 3 | Yes | > 50 | Heifer and Calves | 2 doses first year then annually | 2 | No | NA | 1 |
| Stott 2008 | No | 0 | Yes | 0.30 | 3 | Yes | > 50 | Heifer and Calves | 2 doses first year then annually | 2 | No | NA | 1 |
| Stott 2008 | No | 0 | Yes | 0.87 | 3 | Yes | < 50 | Heifer and Calves | 2 doses first year then annually | 2 | No | NA | 1 |
| Stott 2008 | No | 0 | Yes | 0.93 | 3 | No | NA | None | None | 1 | No | NA | 1 |
| Stott 2008 | No | 0 | Yes | 0.30 | 3 | Yes | < 50 | Heifer and Calves | 2 doses first year then annually | 2 | No | NA | 1 |
| Stott 2008 | No | 0 | Yes | 0.30 | 3 | Yes | > 50 | Heifer and Calves | 2 doses first year then annually | 2 | No | NA | 1 |
| Stott 2008 | No | 0 | Yes | 0.70 | 3 | Yes | > 50 | Heifer and Calves | 2 doses first year then annually | 2 | No | NA | 1 |
| Stott 2008 | No | 0 | Yes | 0.69 | 3 | Yes | < 50 | Heifer and Calves | 2 doses first year then annually | 2 | No | NA | 1 |
| Stott 2008 | No | 0 | Yes | 0.30 | 3 | Yes | < 50 | Heifer and Calves | 2 doses first year then annually | 2 | No | NA | 1 |
| Stott 2008 | No | 0 | Yes | 0.76 | 3 | No | NA | None | None | 1 | No | NA | 1 |
| Stott 2008 | No | 0 | Yes | 0.87 | 3 | Yes | > 50 | Heifer and Calves | 2 doses first year then annually | 2 | No | NA | 1 |
| Stott 2008 | No | 0 | Yes | 0.30 | 3 | Yes | > 50 | Heifer and Calves | 2 doses first year then annually | 2 | No | NA | 1 |
| Stott 2008 | No | 0 | Yes | 0.94 | 3 | Yes | < 50 | Heifer and Calves | 2 doses first year then annually | 2 | No | NA | 1 |
| Stott 2008 | No | 0 | Yes | 0.94 | 3 | No | NA | None | None | 1 | No | NA | 1 |
| Stott 2008 | No | 0 | Yes | 0.30 | 3 | Yes | < 50 | Heifer and Calves | 2 doses first year then annually | 2 | No | NA | 1 |
| Stott 2008 | No | 0 | Yes | 0.74 | 3 | Yes | > 50 | Heifer and Calves | 2 doses first year then annually | 2 | No | NA | 1 |
| Stott 2008 | No | 0 | Yes | 0.30 | 3 | Yes | > 50 | Heifer and Calves | 2 doses first year then annually | 2 | No | NA | 1 |
| Stott 2008 | No | 0 | Yes | 0.90 | 3 | Yes | < 50 | Heifer and Calves | 2 doses first year then annually | 2 | No | NA | 1 |
| Stott 2008 | No | 0 | Yes | 0.94 | 3 | No | NA | None | None | 1 | No | NA | 1 |
| Stott 2008 | No | 0 | Yes | 0.30 | 3 | Yes | < 50 | Heifer and Calves | 2 doses first year then annually | 2 | No | NA | 1 |
| Stott 2003 | No | 0 | Yes | NA | 2 | No | NA | None | None | 1 | Yes | NA | 3 |
| Stott 2003 | No | 0 | Yes | 0.50 | 3 | No | NA | None | None | 1 | Yes | NA | 3 |
| Stott 2003 | No | 0 | Yes | 0.75 | 3 | No | NA | None | None | 1 | Yes | NA | 3 |
| Stott 2003 | No | 0 | Yes | 0.90 | 3 | No | NA | None | None | 1 | Yes | NA | 3 |
| Stott 2003 | No | 0 | Yes | NA | 2 | No | NA | None | None | 1 | Yes | NA | 3 |
| Stott 2003 | No | 0 | Yes | 0.50 | 3 | No | NA | None | None | 1 | Yes | NA | 3 |
| Stott 2003 | No | 0 | Yes | 0.90 | 3 | No | NA | None | None | 1 | Yes | NA | 3 |
| Stott 2003 | No | 0 | Yes | NA | 2 | No | NA | None | None | 1 | Yes | NA | 3 |
| Stott 2012 | No | 0 | No | NA | 1 | Yes | > 50 | Heifer and Calves | 2 doses first year then annually | 2 | No | NA | 1 |
| Stott 2012 | No | 0 | No | NA | 1 | Yes | > 50 | Heifer and Calves | 2 doses first year then annually | 2 | No | NA | 1 |
| Stott 2012 | No | 0 | No | NA | 1 | Yes | > 50 | Heifer and Calves | 2 doses first year then annually | 2 | No | NA | 1 |
| Santman 2015 | Yes | NA | No | NA | 1 | Yes | > 50 | Reproductive females | Annually | 3 | Yes | NA | 3 |
| Santman 2015 | Yes | NA | No | NA | 1 | Yes | > 50 | Reproductive females | Annually | 3 | Yes | NA | 3 |
| Santman 2015 | Yes | NA | No | NA | 1 | Yes | > 50 | Reproductive females | Annually | 3 | Yes | NA | 3 |

**Table S3:** Abbreviation list of the influence case diagnostic shown in Figure S2-S4.

| Abbreviation | Description |
| --- | --- |
| rstudent | Externally standardized residuals |
| dffits | DFFITS values |
| cook.d | Cook’s distances |
| cov.r | Covariance ratios |
| tau2.del | Estimates of τ² when each study is removed in turn |
| QE.del | Test statistics for (residual) heterogeneity when each study is removed in turn |
| hat | Hat values |
| weight | Weights (in %) given to the observed outcomes during the model fitting |

**Table S4:** Full references of the considered studies in the final meta-regression analysis.

Bennett, R.M., Done, J.T., 1986. Control of BVD: a case for social cost-benefit analysis? In:

Proc. of the Meeting of the Society of Veterinary Epidemiology and Preventive Medicine, 2-4 April 1986 Edinburgh, pp. 54-65.

Bitsch, V., Rønsholt, L., 1995. Control of bovine viral diarrhea virus infection without vaccines. [Vet. Clin. North Am. Food Anim. Pract.](http://www.vetfood.theclinics.com/) 11, 627-640.

Chi, J., VanLeeuwen, J.A., Weersink, A., Keefe, G.P., 2002. Direct production losses and treatment costs from bovine viral diarrhoea virus, bovine leukosis virus, *Mycobacterium avium* subspecies *paratuberculosis*, and *Neospora caninum*. Prev. Vet. Med. 55, 137-153.

Dufour, B., Repiquet, D., Touratier, A., 1999. Place des études économiques dans les

decisions de santé animale: exemple du rapport cout/bénéfice de l'éradication de la

diarrhée viral bovine en France. Rev. Sci. et Tech. Off. Int. Epiz. 18, 520–532.

Gunn, G.J., Stott, A.W., Humphry, R.W., 2004. Modelling and costing BVD outbreaks in

beef herds. Vet. J. 167, 143-149.

Häsler, B., Howe, K.S., Presi, P., Stärk, K.D.C., 2012. An economic model to evaluate the

mitigation program for bovine viral diarrhoea in Switzerland. Prev. Vet. Med. 106, 162–173.

Larson, R.L., Pierce, V.L., Grotelueschen, D.M., Wittum, T.E., 2002. Economic evaluation of

beef cow herd screening for cattle persistently-infected with bovine viral diarrhea virus. Bov. Pract. 36, 106-112.

Marschik, T., Obritzhauser, W., Wagner, P., Richter, V., Mayerhofer, M., Egger-Danner,C.,

Käsbohrer,A., Pinior, B., 2018. A cost-benefit analysis and the potential trade effects of the bovine viral diarrhoea eradication programme in Styria, Austria. Vet. J. 231, 19-29.

Pasman, E.J., Dijkhuizen, A.A., Wentink, G.H., 1994. A state-transition model to simulate the economics of bovine virus diarrhea control. Prev. Vet. Med. 20, 269-277.

Reichel, M.P., Hill, F.I., Voges, H., 2008. Does control of bovine viral diarrhoea infection make economic sense? N. Z. Vet. J. 56, 60-66.

Santman-Berends, I.M.G.A., Mars, M.H., van Duijn, L., van Schaik, G., 2015. Evaluation of the epidemiological and economic consequences of control scenarios for bovine viral diarrhea virus in dairy herds. J. Dairy Sci. 98, 7699-7716.

Smith, R.L., Sanderson, M.W., Jones, R., N’Guessan, Y., Renter, D., Larson, R., White, B.J.,

2014. Economic risk analysis model for bovine viral diarrhea virus biosecurity in cow-calf herds. Prev. Vet. Med. 113, 492–503.

Stott, A.W., Lloyd, J., Humphry, R.W., Gunn, G.J., 2003. A linear programing approach to

estimate the economic impact of bovine viral diarrhoea (BVD) at the whole-farm level

in Scotland. Prev. Vet. Med. 59, 51–66.

Stott, A.W., Gunn, G.J., 2008. Use of a benefit function to assess the relative investment

potential of alternative farm animal disease prevention strategies. Prev. Vet. Med. 84, 179-193.

Stott, A.W., Humphry, R.W., Gunn, G.J., 2010. Modelling the effects of previous infection

and re-infection on the costs of bovine viral diarrhoea outbreaks in beef herds. Vet. J. 185, 138-143.

Stott, A.W., Humphry, R.W., Gunn, G.J., Higgins, I., Hennessy, T., O'Flaherty, J., Graham,

D.A., 2012. Predicted costs and benefits of eradicating BVDV from Ireland. Ir. Vet. J. 65, 12. doi:10.1186/2046-0481-65-12

Thomann, B., Tschopp, A., Magouras, I., Meylan, M., Schüpbach-Regula, G., Häsler, B.,

2017. Economic evaluation of the eradication program for bovine viral

diarrhea in the Swiss dairy sector. Prev. Vet. Med. 145, 1-6.

Valle, P.S., Skjerve, E., Martin, S.W., Larssen, R.B., Østerås, O., Nyberg, O., 2005. Ten

years of bovine virus diarrhoea virus (BVDV) control in Norway: a cost-benefit analysis. Prev. Vet. Med. 72, 189-207.

Weldegebriel, H.T., Gunn, G.J., Stott, A.W., 2009. Evaluation of producer and consumer

benefit resulting from eradication of bovine viral diarrhoea (BVD) in Scotland, United Kingdom. Prev. Vet. Med. 88, 49-56.
